# Activation of Plant Innate Immunity by Extracellular High Mobility Group Box 3 and Its Inhibition by Salicylic Acid

**DOI:** 10.1371/journal.ppat.1005518

**Published:** 2016-03-23

**Authors:** Hyong Woo Choi, Murli Manohar, Patricia Manosalva, Miaoying Tian, Magali Moreau, Daniel F. Klessig

**Affiliations:** Boyce Thompson Institute for Plant Research, Ithaca, New York, United States of America; University of California, Davis Genome Center, UNITED STATES

## Abstract

Damage-associated molecular pattern molecules (DAMPs) signal the presence of tissue damage to induce immune responses in plants and animals. Here, we report that High Mobility Group Box 3 (HMGB3) is a novel plant DAMP. Extracellular HMGB3, through receptor-like kinases BAK1 and BKK1, induced hallmark innate immune responses, including i) MAPK activation, ii) defense-related gene expression, iii) callose deposition, and iv) enhanced resistance to *Botrytis cinerea*. Infection by necrotrophic *B*. *cinerea* released HMGB3 into the extracellular space (apoplast). Silencing *HMGB*s enhanced susceptibility to *B*. *cinerea*, while HMGB3 injection into apoplast restored resistance. Like its human counterpart, HMGB3 binds salicylic acid (SA), which results in inhibition of its DAMP activity. An SA-binding site mutant of HMGB3 retained its DAMP activity, which was no longer inhibited by SA, consistent with its reduced SA-binding activity. These results provide cross-kingdom evidence that HMGB proteins function as DAMPs and that SA is their conserved inhibitor.

## Author Summary

In mammals, extracellular HMGB1 is the prototypic Damage-Associated Molecular Pattern (DAMP) molecule, which activates inflammatory and immune responses to protect against infection and promote healing after tissue damage. Increasing evidence argues that it also plays important roles in many diseases. In contrast, the role of HMGB proteins in plant immunity has not been reported. We recently identified human HMGB1 as a novel Salicylic Acid-Binding Protein (SABP) and found that its DAMP activities are specifically inhibited by SA binding. In this study, we showed that i) infection by a necrotrophic pathogen releases plant HMGB3 into the apoplast, ii) extracellular, HMGB3 activates immune responses, iii) SA binds to HMGB3, and iv) this binding alters its DAMP activity. These findings provide the first demonstration that a plant HMGB function as a DAMP, like its human counterpart, as well as the insights into how SA inhibits both plant and animal HMGB-induced immunity.

## Introduction

As multi-cellular organisms, plants must be able to detect wounding and tissue damage, which can breach the physical barriers to pathogen infection. Damage-associated molecular patterns (DAMPs; also known as “danger signals” or “alarmins”) are endogenous molecules that are constitutively present; they are released into the extracellular space in response to stress or tissue damage to elicit responses that promote wound healing and prime the surrounding tissue for future injury or infection [1–3]. Cell surface pattern-recognition receptor (PRR) complexes detect the presence of DAMPs, as well as microbe-associated molecular patterns (MAMPs), and activate a plant innate immune response termed pattern-triggered immunity (PTI) [1,3,4]. DAMPs seem to be evolutionary conserved within the plant kingdom, with some, such as oligogalacturonides (OGs), present throughout the kingdom, while others, such as the Peps, present in only certain plant species [1,5,6].

To date, three different types of DAMPs and their cognate PRRs have been identified in plants: Pep1 and Pep2, derived from ProPep precursor polypeptide, and their receptors PEPR1 and PEPR2 [7–9], Wall-Associated Kinase WAK1 for OGs [10,11] and Does Not Respond to Nucleotides 1 (DORN1) for extracellular ATP (eATP) [12]. Notably, several Arabidopsis PRRs, which belong to the ligand-binding, leucine-rich repeat receptor kinase (LRR RK) class, including PEPR1, PEPR2, the EF-Tu receptor EFR, and the flagellin receptor FLS2, share the same regulatory LRR receptor-like kinases (LRR RLKs), BRI1-Associated receptor Kinase 1 (BAK1) and BAK1-LIKE Kinase 1 (BKK1), as signaling partners [13–16]. Plants lacking certain PRRs or their regulatory LRR RLKs BAK1 and BKK1 have severely compromised PTI responses. These plants fail to exhibit a rapid Ca^2+^ influx or an oxidative burst, and/or display reduced levels of mitogen-activated protein kinase (MAPK) activation, defense-related gene expression, and callose deposition; as a result, they exhibit decreased disease resistance.

In mammals, a number of different DAMPs have been characterized, including Heat Shock Proteins (HSPs), eATP, uric acid, mitochondrial DNA, cholesterol, and High Mobility Group Box 1 (HMGB1) [2]. These molecules are passively released into the extracellular milieu by damaged or dying cells or can be actively secreted by immune or severely stressed cells, such as certain cancer cells. Extracellular DAMPs are recognized by their cognate PRR receptors, which then activate intracellular signaling pathways that involve, for example, MAPK activation and expression of a subset of pro-inflammatory genes.

Mammalian HMGB1 was one of the first DAMPs to be identified and extensively characterized; hence, it is considered a prototypic DAMP. Human HMGB1 (hereafter hHMGB1) has attracted considerable attention due to its potent pro-inflammatory activities associated with diverse and major human diseases, such as certain types of cancers (colorectal, gastric, breast and pancreatic cancers, melanoma and mesothelioma), as well as sepsis, lupus, arthritis, atherosclerosis, and ischemia and reperfusion (I/R) injury [17–19]. In the nucleus, hHMGB1 binds the minor groove of DNA to facilitate nucleosome formation and transcription factor binding [17,20]. Upon its release into the extracellular milieu, it is recognized by various cell surface receptors, including Receptor for Advanced Glycation Endproducts (RAGE), Toll-Like Receptor 2 (TLR2), TLR4, and C-X-C chemokine Receptor type 4 (CXCR4), which likely accounts for its multiple roles in disease [19,21–23]. Recently, our group reported that salicylic acid (SA), which is a critical immune-regulating hormone in plants, as well as the primary metabolite of aspirin (acetyl salicylic acid) in humans, inhibits hHMGB1’s DAMP activity by direct interaction with residues Arg24 and Lys28 of its HMG box domain [24].

All eukaryotic cells, including plants, have hHMGB1-related proteins. In Arabidopsis, 15 genes encoding HMG-box domain-containing proteins have been identified. They can be subdivided into four groups: (i) HMGB-type proteins, (ii) A/T-rich interaction domain (ARID)-HMG proteins, (iii) 3xHMG proteins that contain three HMG boxes, and (iv) the structure-specific recognition protein 1 (SSRP1) [25]. Based on their nuclear location, the eight HMGB-type proteins (HMGB1/2/3/4/5/6/12/14) are proposed to function as architectural chromosomal proteins, like hHMGB1. While the majority of these proteins are found exclusively in the nucleus, HMGB2/3/4 are also present in the cytoplasm [25–27]. At present their function, if any, in the cytoplasm is unclear.

Here, we demonstrate that extracellular Arabidopsis HMGB3 exhibits DAMP-like activities, similar to those of the well-characterized plant DAMP, Pep1 peptide since both induced MAPK activation, callose deposition, defense-related gene expression, and enhanced resistance. Biochemical and molecular genetic evidence indicates that HMGB3 interacts with SA using conserved Arg and Lys residues in the HMG box, which enables SA inhibition of its DAMP activity. Together, these findings provide the first demonstration of HMGB function as a DAMP in plant innate immunity, as well as key insights into how SA inhibits both plant and animal HMGB-mediated PTI.

## Results

### Extracellular HMGB3 activates innate immune responses and induces disease resistance

Since HMGB3 is present in the cytoplasm as well as the nucleus, it has greater access to the extracellular space (apoplast) after cellular damage compared to the HMGBs located exclusively in the nucleus (e.g. HMGB1 and HMGB5) [25–27] because the cytoplasmic subpopulation is not bound to DNA and need only cross the plasma membrane system to enter the apoplast. HMGB3’s location, together with the well-established role of mammalian HMGB1 as the prototypic DAMP [17], suggested that it might also function as a DAMP. To test this possibility, purified endotoxin-free *E*. *coli*-expressed recombinant HMGB3 and the positive control synthetic Pep1 were infiltrated into the extracellular space of Arabidopsis leaves and early immune responses activated during PTI were monitored. His-tagged recombinant maltose-binding protein (MBP), purified in parallel with HMGB3, served as a negative control to insure that induction of immune responses by recombinant HMGB3 preparations was not due to a contaminating bacterial elicitor-active molecules since such a contaminant would also be present in the recombinant MBP preparation.

**Fig 1:**
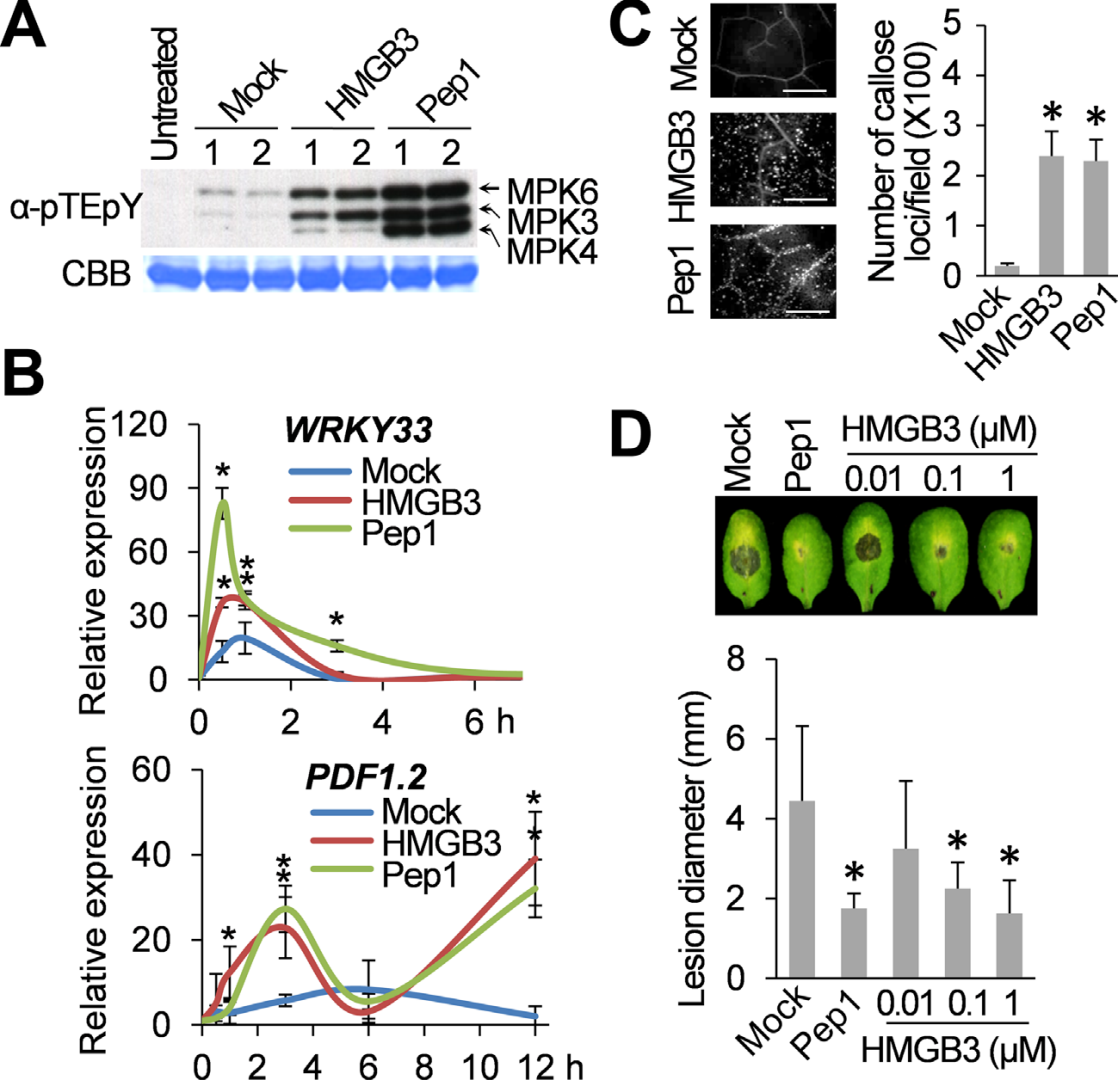
Extracellular HMGB3 activates innate immune responses. **A.** HMGB3- or Pep1-induced MAPK activation in Arabidopsis. Leaves were collected 15 min after infiltration with water containing either 1 μM recombinant HMGB3 (HMGB3) or Pep1 peptide (Pep1) for the MAPK activation assay. Activation of MPK3, MPK4, and MPK6 by MAPK kinase-mediated phosphorylation of the TEY sequence was detected with α-pTEpY antibody using immune-blot (IB) analyses. Ribulose-1,5-bisphosphate carboxylase/oxygenase (Rubisco) large subunit protein stained with Coomassie Brilliant Blue (CBB) served as a loading control. 1 and 2 denote independent biological replicates. **B.** RT-PCR analysis of *WRKY33* (upper panel) and *PDF1*.*2* (lower panel) expression at the indicated times after infiltration with water containing either 0.1 μM HMGB3 or Pep1. Expression levels were plotted relative to the expression in untreated leaves. Data are the mean ± SD (n = 4). **C.** HMGB3- or Pep1-induced callose deposition in Arabidopsis. Leaves were stained with aniline blue 15 h after infiltration with water containing either 0.1 μM HMGB3 or Pep1. Representative pictures are shown in the left panel. Bars = 100 μm. Data are the mean ± SD (right panel, n = 20). **D.** HMGB3- or Pep1-induced resistance to *B*. *cinerea*. Leaves were infiltrated with water containing the indicated concentrations of HMGB3 or 1 μM Pep1 one day before *B*. *cinerea* inoculation. Representative disease symptoms at 3 days post infection (dpi) are shown in the upper panel. Data corresponding to this time point are presented as the mean ± SD (lower panel, n = 6). Leaves infiltrated with water served as mock control in all experiments. Asterisks in **B**, **C**, and **D** indicate significant differences from the mock-treated leaves (*t* test, *P* < 0.05).

Initially, we assessed the activation of MPK3 and MPK6, which are orthologs of tobacco wound-induced protein kinase and SA-induced protein kinase, respectively, and MPK4 [16,28,29]. MAPK activation was monitored 15 min after infiltration of HMGB3, MBP, or Pep1 using an anti-pTE-pY antibody, which detects phosphorylation of the TEY motif by upstream MAPK kinases, resulting in MAPK activation [16]. Infiltrated HMGB3 or Pep1, but not MBP, rapidly activated MPK3 and MPK6, although the activation by HMGB3 was not as strong as that induced by Pep1 (**Fig 1A and S1A Fig**). Pep1 also strongly activated MPK4, which negatively regulates immune responses under some circumstances [29,30]. The ability of HMGB3 to induce the expression of *WRKY33* and *PDF1*.*2*, two genes known to be induced by Pep1 [9], was then tested. The induction patterns for *WRKY33* and *PDF1*.*2* following HMGB3 infiltration mirrored those observed after Pep1 treatment (**Fig 1B**). For example, *PDF1*.*2* mRNA exhibited a biphasic accumulation pattern after HMGB3 or Pep1 treatment, but not following mock treatment. Callose deposition, another important immune-related response that is frequently used to assess PTI [3,31], also was strongly induced by both HMGB3 and Pep1 (**Fig 1C**). In contrast, the negative control MBP preparation failed to induce callose deposition (**S1B Fig**); thus, indicating that the induction immune responses was due to HMGB3 rather than to an *E*. *coli-*derived contaminant. Finally, we assessed whether infiltration of HMGB3 provides protection against the necrotrophic fungal pathogen *B*. *cinerea*. Indeed, HMGB3 enhanced resistance to this pathogen in a dose-dependent manner, with 1 μM HMGB3 providing comparable levels of protection as 1 μM Pep1 (**Fig 1D**). The amount of suppression of fungal growth by infiltrated HMGB3 was estimated based on the reduction in lesion size (**Fig 1D**) and confirmed by semi-quantitative RT-qPCR (**S2 Fig**). Together, these results indicate that extracellular HMGB3 induces Arabidopsis innate immune responses and enhances resistance, similar to the well-studied plant DAMP Pep1.

### *BAK1* and *BKK1* are required for extracellular HMGB3-induced innate immune responses

**Fig 2:**
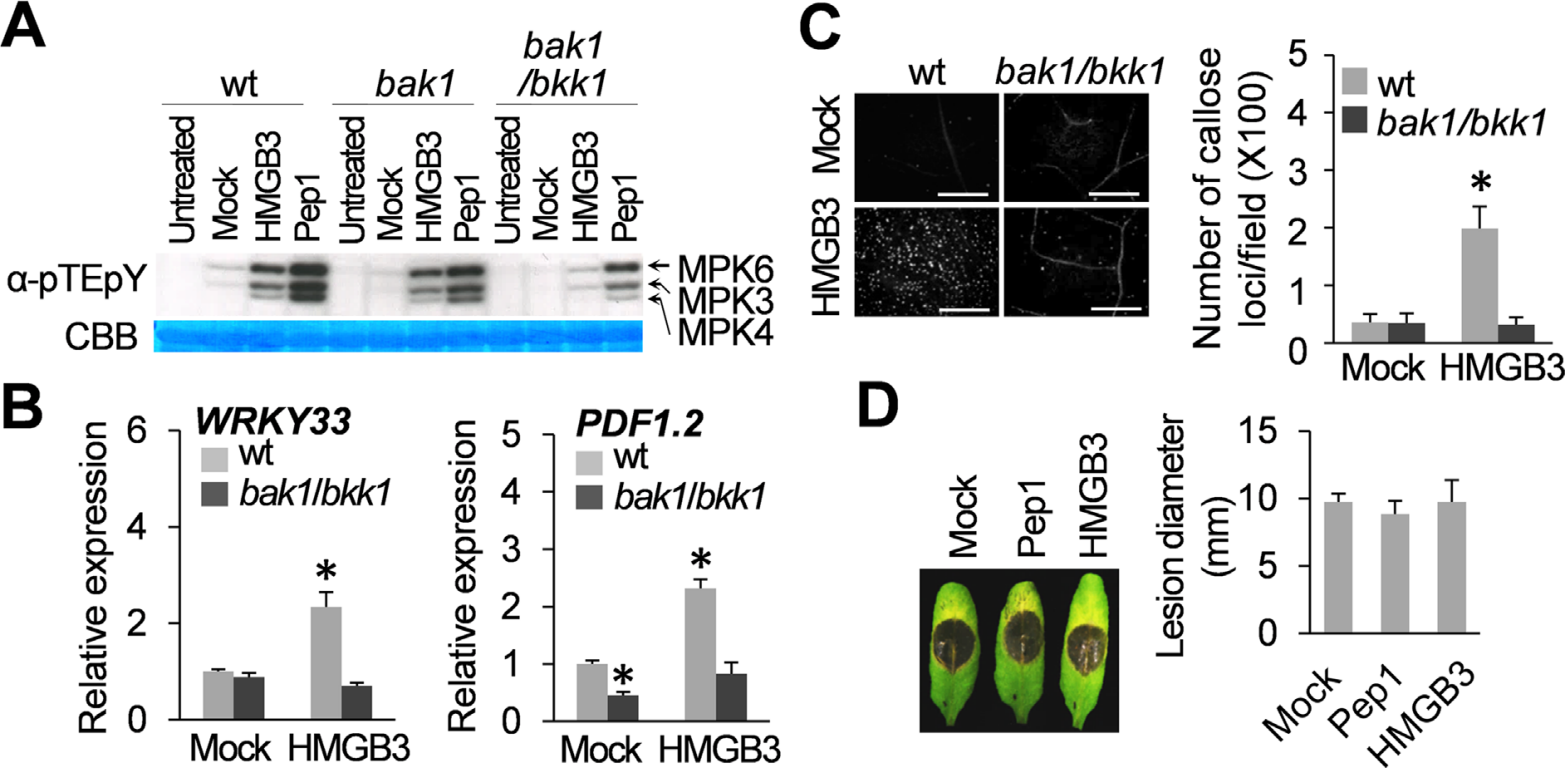
BAK1 and BKK1 are required for extracellular HMGB3-induced innate immune responses. **A.** HMGB3- or Pep1-induced MAPK activation was compromised in *bak1-5* and *bak1-5/bkk1-1*. Leaves were collected 15 min after infiltration with water containing 1 μM HMGB3 or Pep1 for the MAPK activation assay. pTEpY phosphorylation of the TEY motif of MPK3, MPK4, and MPK6 was detected with α-pTEpY antibody. Rubisco large subunit protein stained with CBB served as the loading control. **B.** HMGB3-induced defense-related gene expression was compromised in *bak1-5/bkk1-1*. Leaves were collected 30 min after infiltration with water containing 0.1 μM HMGB3. Following RT-PCR, expression levels were plotted relative to the expression in water-treated (mock) wt leaves, which was set at 1. Data are the mean ± SD (n = 4). **C.** HMGB3-induced callose deposition was compromised in *bak1-5/bkk1-1*. Leaves were stained with aniline blue 15 h after infiltration with water containing 1 μM HMGB3. Representative pictures are shown in the left panel. Bars = 100 μm. Data are the mean ± SD (right panel, n = 20). **D.** HMGB3- or Pep1-induced resistance to *B*. *cinerea* was compromised in *bak1-5/bkk1-1*. Leaves were infiltrated with water containing 1 μM HMGB3 or Pep1 one day before *B*. *cinerea* infection. Representative disease symptoms at 3 dpi are shown in the left panel and the data corresponding to this time point are presented as the mean ± SD (right panel, n = 6). Leaves infiltrated with water served as mock control in experiments **B**-**D**. Asterisks in **B** and **C** indicate significant differences from the mock-treated leaves or between HMGB3-treated wt vs *bak1/bkk1* (*t* test, *P* < 0.05).

PTI signaling triggered by diverse PRRs often requires BAK1 and BKK1 [14]. Thus, we tested whether HMGB3, as well as Pep1, could induce MAPK activation in the *bak1-5* (*bak1*) and *bak1-5*/*bkk1-1* (*bak1/bkk1*) mutant backgrounds [16]. MAPK activation in *bak1* single mutants treated with HMGB3 or Pep1 was moderately reduced compared to that in wild type (wt) plants (25.9% and 14.5% reduction in HMGB3- and Pep1-treated samples, respectively; **Fig 2A**). Activation by both was more drastically reduced in *bak1*/*bkk1* (71.8% and 56.4% reduction in HMGB3- and Pep1-treated samples, respectively), suggesting that HMGB3- and Pep1-induced MAPK activation utilizes the same regulatory LRR RLKs. The ability of HMGB3 to induce the expression of several members of the *WRKY* family of defense-related transcription factors, and also the prototypic jasmonic acid (JA)/ethylene-responsive *PDF1*.*2* gene and the prototypic SA-responsive *PR-1* and *PR-2* genes, was then assessed in *bak1*/*bkk1*. HMGB3-mediated induction of *WRKY33*, *WRKY53*, and *PDF1*.*2* expression was completely suppressed in the *bak1*/*bkk1* mutant as compared with wt plants, whereas that of *WRKY70*, *PR-1*, and *PR-2* was reduced, but still significantly greater than that detected in mock-treated wt plants (**Fig 2B** and **S3 Fig**). This latter result suggests that HMGB3 induces the expression of some defense-related genes via both BAK1/BKK1-independent and -dependent pathways. HMGB3-induced callose deposition also was impaired in *bak1*/*bkk1* plants, as there was no significant difference between mock-treated wt and HMGB3-treated *bak1*/*bkk1* plants (**Fig 2C**). Importantly, the ability of HMGB3, as well as Pep1, to enhance resistance to *B*. *cinerea* was completely compromised in *bak1*/*bkk1* (**Fig 2D**). Consistent with an earlier report [32], *bak1*/*bkk1* plants exhibited greater susceptibility compared to wt plants as shown by increased lesion diameter (compare **Fig 1D** and **Fig 2D**).

### *B*. *cinerea* infection release HMGB3 into the apoplast

**Fig 3:**
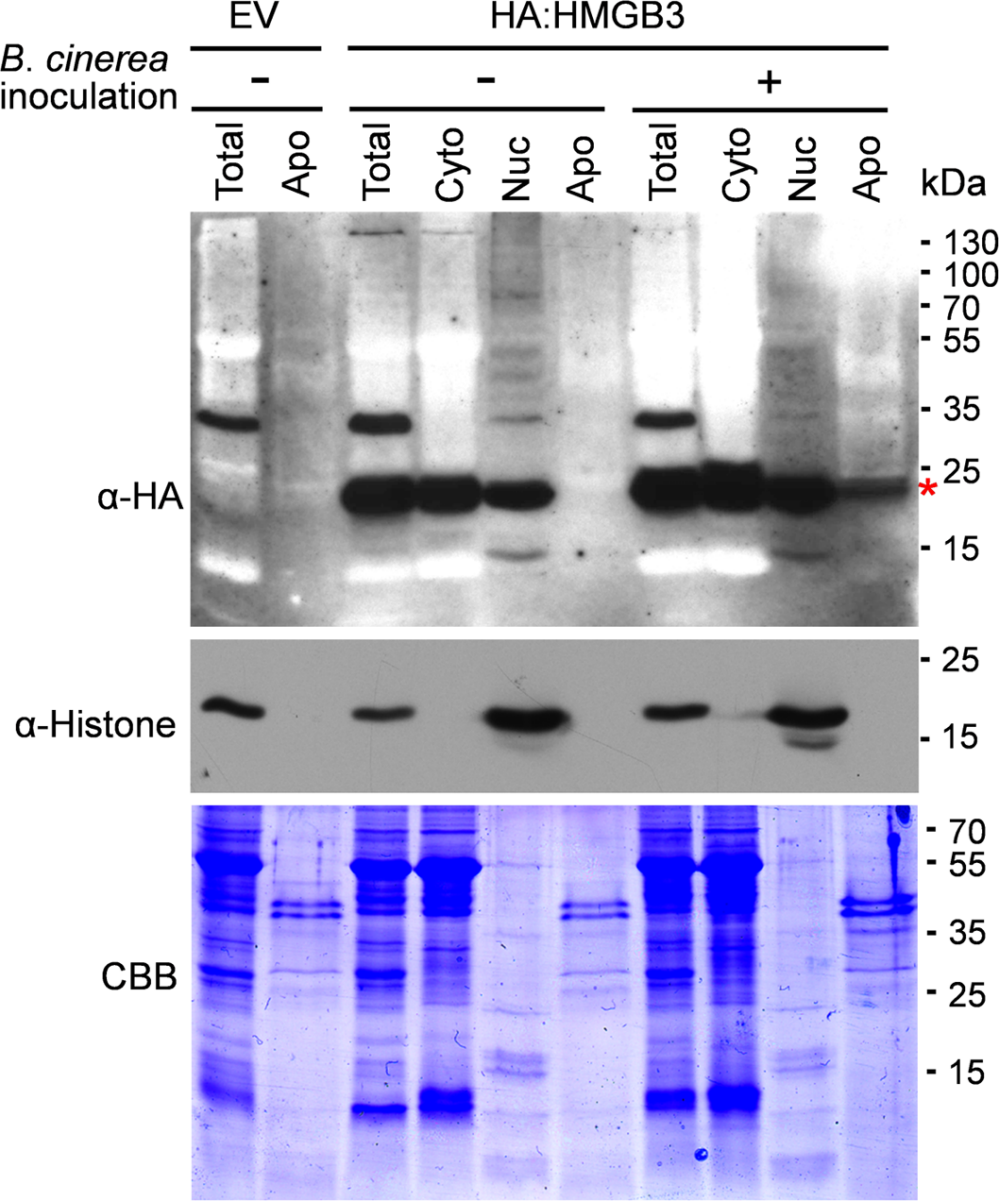
HMGB3 is released into apoplast by *B*. *cinerea* infection. HA-HMGB3 was detected in apoplast only after *B*. *cinerea* inoculation. HMGB3 localized in nucleus and cytoplasm, but not in apoplast, in the absence of the pathogen. Total leaf extract, the apoplastic fraction (Apo), cytoplasmic (Cyto), and nuclear (Nuc) fractions were size fractionated on 12% SDS-PAGE followed by IB analyses with α-HA or α-histone H3 antibody. Histone H3 level were monitored to assess contamination by nuclear proteins. *N*. *benthamiana* leaves were spray inoculated with a spore suspension of *B*. *cinerea* one day after infection with Agrobacterium carrying an empty vector (EV) or the HA:HMGB3 expression vector. Total and subcellular protein fractions were prepared one day after *B*. *cinerea* inoculation (two days after agro-infection). The band detected at the expected size for HA-HMGB3 (~20 kDa) is marked with red asterisk. CBB stained gel indicates that loading of three apoplastic fractions were very similar, as was loading of the two cytoplasmic fractions and the two nuclear fractions.

In animal systems HMGB1 is passively released from necrotic or damaged cells into the extracellular milieu, which induces immune responses, including inflammation [17–19]. To assess whether HMGB3 is similarly released into the apoplast from necrotic cells, we utilized the *Nicotiana benthamiana* transient expression system, since HMGB3-specific antibodies are not available. Using Agrobacterium-mediated gene transfer HA-tagged HMGB3 under the constitutive CaMV 35S promoter was expressed in *N*. *benthamiana* leaves. One day later a portion of the infected leaves were inoculated with *B*. *cinerea*, which cause necrosis. As a negative control Agrobacterium carrying an empty vector was used in parallel. Apoplastic fluid, total leaf tissue extract, and cytoplasmic and nuclear fractions were prepared one day later and analysed by immunoblotting (IB) using α-HA or α-histone antibodies. A specific band at the expected size (~20 kDa) was detected with the α-HA antibody in total leaf extracts and in the cytoplasmic and nuclear fractions of leaves expressing HA-HMGB3, but not from leaves expressing the empty vector (EV; **Fig 3**). In the absence of *B*. *cinerea* inoculation HMGB3 was not detected in the apoplastic fraction. In contrast, it was readily detected in the apoplastic fraction prepared from *B*. *cinerea*-inoculated leaves, indicating that HMGB3 can be released into apoplast during pathogen-induced necrosis.

As a control for fractionation we monitored histone H3 (H3). As expected H3 was present only in the total leaf extracts and nuclear fractions in *B*. *cinerea* inoculated as well as uninoculated leaves. The absence of H3 in the cytoplasmic and apoplastic fractions one day after inoculation is consistent with the lack of visible necrosis and suggest that only mild necrosis had occurred at this early stage of infection. The substantial amount of HMBG3 already present in the apoplast within 24 hour post inoculation suggest that its release into the apoplast occurs early in cellular necrosis and thus could enhance resistance by inducing immune responses.

### Silencing of *HMGB*s confers increased susceptibility to *B*. *cinerea*

**Fig 4:**
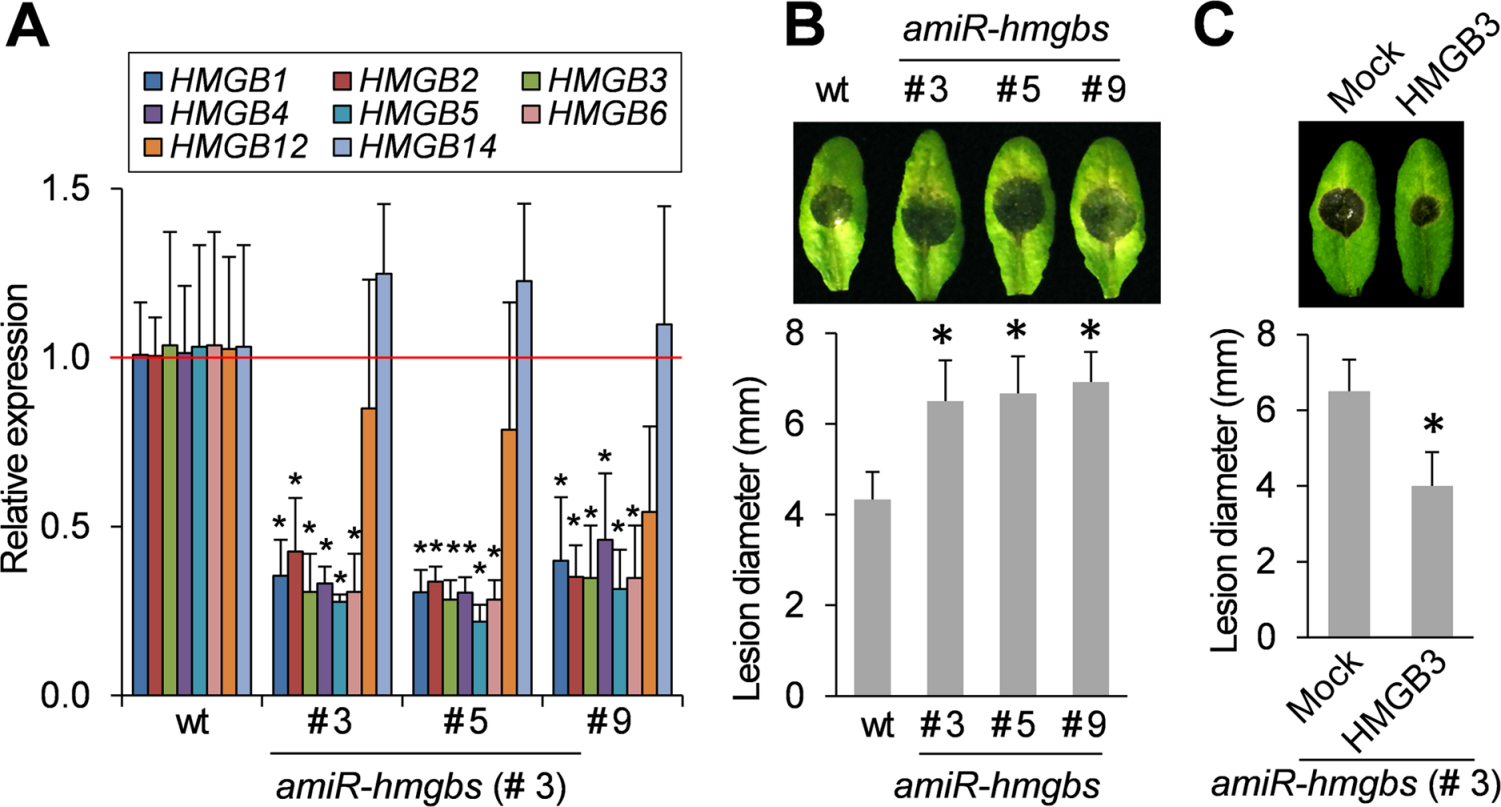
Silencing *HMGB*s increases susceptibility to *B*. *cinerea*. **A.** Relative expression of *HMGBs* (*HMGB1*-*HMGB14*) in 4-week-old plant from three different *amiR-hmgbs* lines (#3, #5 and #9) was compared to their expression levels in wt plants, which was set at 1 and denoted by a red line. Data are the mean ± SD (n = 4). **B.** *amiR-hmgbs* lines showed enhanced susceptibility to *B*. *cinerea* infection. Representative disease symptoms at 3 dpi are shown in the upper panel, and the data corresponding to this time point are presented in the lower panel as the mean ± SD (n = 6). **C.** Extracellular HMGB3 restored resistance of *amiR*-*hmgbs* transgenic plants (line # 3) to *B*. *cinerea*. Leaves were infiltrated with water containing 1 μM HMGB3 one day before *B*. *cinerea* infection. Representative disease symptoms at 3 dpi are shown in the upper panel. Data corresponding to this time point are presented as the mean ± SD (lower panel, n = 6). Asterisks indicate significant differences from the untransformed wt plants in **A** and **B** or mock-treated *amiR*-*hmgbs* transgenic plants in **C** (*t* test, *P* < 0.05).

Previous studies have indicated that Arabidopsis mutants deficient in certain *HMGB*s, such as *HMGB1* or *HMGB5*, exhibit rather mild, if any, phenotypic changes under optimal growth conditions, but show reduced tolerance to abiotic stress treatment [33–35]. However, little is known about HMGB’s involvement in plant immunity. To address this question, we generated *HMGB*-silenced Arabidopsis plants by overexpressing an artificial microRNA (hereafter, *amiR-hmgbs*) designed to target *HMGB3*. Due to the high sequence similarity the designed *amiR-hmgbs was* predicted to also target HMGB2 and HMGB1, which encodes an exclusively nuclear HMGB [26] (**S4A Fig**). Three different lines (#3, #5 and #5) showing significantly reduced mRNA levels for *HMGB1/2/3* were selected for further analyses (**Fig 4A**). Unexpectedly, transcript levels for *HMGB4*/*5*/*6* also were reduced in all three *amiR-hmgbs* lines. Nucleotide (nt) sequence alignment of *amiR-hmgbs* with the predicted target region on *HMGB*4/5/6 revealed that *HMGB4* has the same degree of identity with *amiR-hmgbs* as *HMGB1*/*2* (17nt/21nt: ~81% identity, compared to 91% identity for HMGB3, with 19 of 21 nts matching). Lesser degrees of identity were observed for *HMGB5* (~67% identity) and *HMGB6* (~62% identity) (**S4B Fig).** This analysis suggests that *HMGB4* is a likely target of *amiR*-*hmgbs*, while the expression of *HMGB5*/*6* may be indirectly down regulated in *amiR-hmgbs* plants via an unknown mechanism.

Soil-grown *amiR-hmgbs* plants showed no aberrant developmental phenotype compared to untransformed wt plants. In addition, untreated *amiR-hmgbs* plants did not display altered expression of *PR-1* or *PDF1*.*2* (**S5 Fig**). However, the *amiR-hmgbs* plants showed significantly greater susceptibility to *B*. *cinerea* than wt plants (**Fig 4B**), suggesting that *HMGB*s are required for basal resistance to this fungal pathogen. To confirm that this increased susceptibility was due to decreased HMGB expression, purifed HMGB3 was infiltrated into *amiR-HMGBs* line #3 one day before infection with *B*. *cinerea* (**Fig 4C**). HMGB3-infiltrated leaves developed significantly smaller necrotic lesions than mock-treated leaves, indicating that extracellular HMGB3 can reverse the enhanced susceptibility of the *amiR-hmgbs* plants. This result also suggest that the enhanced susceptibility in the HMGB-silenced plants was due, at least in part, to reduced defense signaling by endogenous extracellular HMGB3.

### SA binds to HMGB3 and inhibits it’s immune-inducing activities

**Fig 5:**
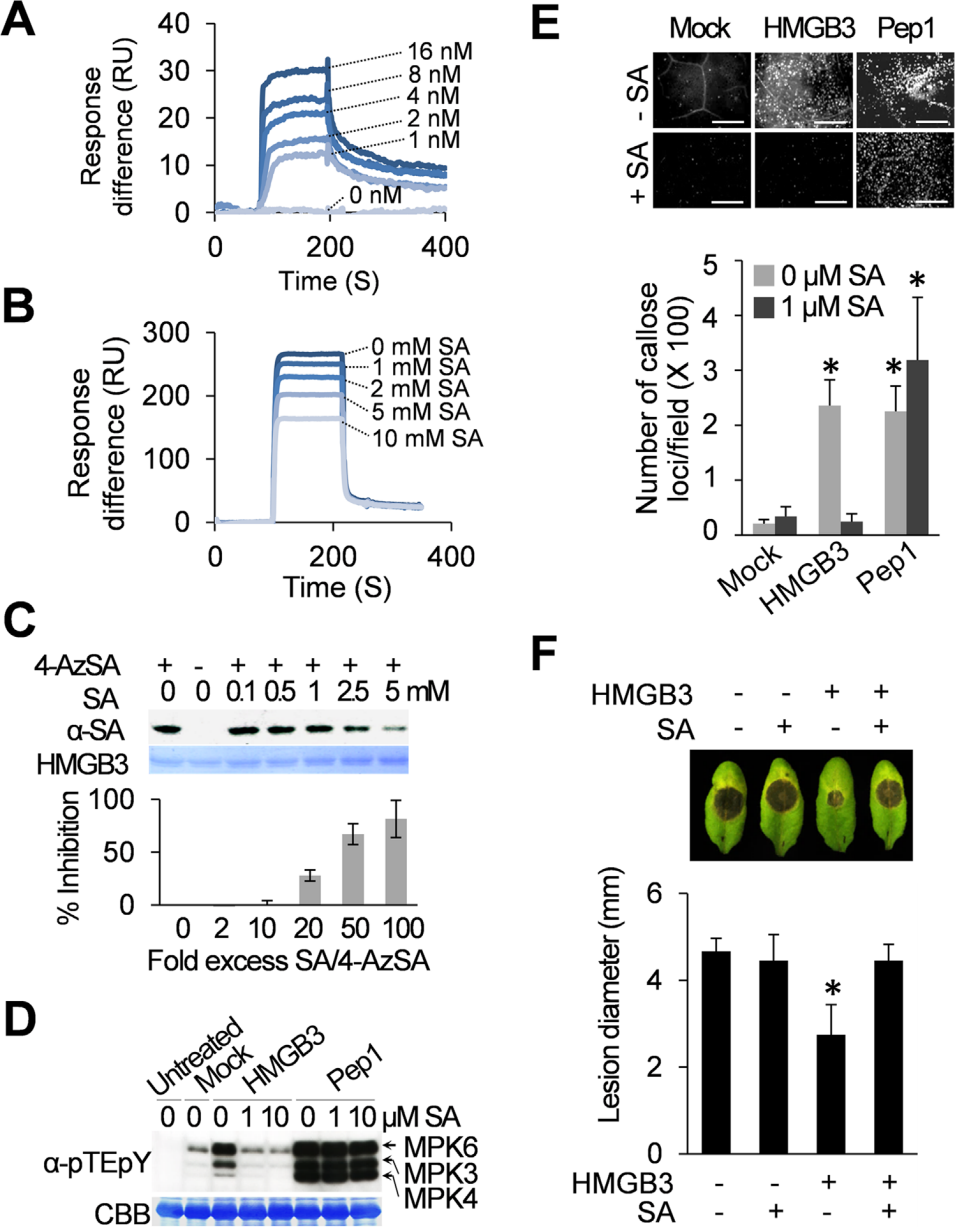
SA binds to HMGB3 and inhibits its DAMP activity. **A**-**B.** SPR analysis of HMGB3’s SA-binding activity. **A.** Sensorgrams obtained with different concentrations of HMGB3 using a 3AESA-immobilized sensor chip. **B.** Sensorgrams obtained with 1 μM HMGB3 in the presence of indicated concentrations of SA using a 3AESA-immobilized chip. The signals detected from a mock-coupled control chip were subtracted. **C.** Photoaffinity labeling of HMGB3 using 4-AzSA. HMGB3 was incubated with 50 μM 4-AzSA in the presence of different concentrations of SA, and then exposed to UV light (30 mJ). HMGB3 labeled with 4-AzSA was detected by IB analysis with α-SA antibodies. HMGB3 stained with CBB served as a loading control. The results are expressed as a percentage of inhibition in the presence of the indicated fold excess of SA as compared to the amount 4-AzSA crosslinked HMGB3 formed in the absence of SA, which was assigned 0% inhibition. Data are the mean ± SD (n = 2). **D.** SA inhibited HMGB3-induced, but not Pep1-induced, MAPK activation. Leaves were collected 15 min after infiltration with water (mock) or with water containing either 1 μM HMGB3 or Pep1 in the presence of the indicated concentrations of SA. Leaves not infiltrated served as an untreated control. Phosphorylated, and thus activated, of MPK3, MPK4, and MPK6 were detected with α-pTEpY antibody. Rubisco large subunit protein stained with CBB served as a loading control. **E.** SA inhibited HMGB3’s, but not Pep1’s, ability to induce callose deposition. Leaves were stained with aniline blue 15 h after infiltration with water containing 0.1 μM HMGB3 or Pep1 in the presence or absence of 1 μM SA. Representative pictures are shown in upper panel. Bars = 100 μm. Data are the mean ± SD (lower panel, n = 20). **F.** SA inhibited enhanced resistance to *B*. *cinerea* induced by HMGB3. Leaves were infiltrated with water containing 100 nM HMGB3 with or without 1 μM SA one day before *B*. *cinerea* infection. Representative disease symptoms at 3 dpi are shown in the upper panel. Data corresponding to this time point are presented as the mean ± SD (lower panel, n = 6). Asterisks indicate a significant difference from the mock-treated leaves (*t* test, *P* < 0.05). Leaves infiltrated with water served as controls in experiments **D**-**F**.

Recently, we reported that SA interacts with the HMG box domain of human HMGB1 (hHMGB1), thereby suppressing its pro-inflammatory activities [24]. To determine whether HMGB3 is similarly regulated by SA, we first tested whether it exhibits SA binding activity using surface plasmon resonance (SPR) [36,37]. HMGB3 showed dose-dependent binding to the SA derivative 3-aminoethyl SA (3AESA), which was immobilized on an SPR sensor chip (**Fig 5A**). Kinetic analysis indicated that HMGB3 has very high affinity for this SA derivative with a Kd of 1.5 nM. Additionally, this binding was competed in the presence of increasing concentrations of SA, which argues that this binding represents authentic SA-binding activity (**Fig 5B**). HMGB3’s SA-binding activity was confirmed using photoaffinity crosslinking to 4-azido SA (4AzSA) [36–38]. HMGB3 was effectively crosslinked to 4AzSA by UV irradiation, and the HMGB3-4AzSA complex was detected by immunoblotting with an α-SA antibody (**Fig 5C**). Importantly, the presence of increasing concentrations of SA in the binding/crosslinking reaction also effectively inhibited HMGB3-4AzSA complex formation in a dose-dependent manner, further confirming that HMGB3, like hHMGB1, has authentic SA-binding activity.

Since the pro-inflammatory activities of hHMGB1 are inhibited by its specific interaction with SA [24], we determined whether SA also inhibits HMGB3’s ability to induce PTI-associated defense responses. Co-infiltration of SA completely suppressed HMGB3-induced, but not Pep1-induced, MAPK activation and callose deposition (**Fig 5D** and **5E**). This suggests that co-infiltrated SA specifically inhibits immune responses induced by HMGB3, rather than suppressing all PTI signaling *per se*. Co-infiltrated SA also blocked HMGB3-induced resistance to *B*. *cinerea* (**Fig 5F**). To analyze the effect of endogenous SA on HMGB3’s DAMP activity, we tested extracellular HMGB3-mediated PTI responses in the SA-deficient mutant *sid2*, which is unable to synthesize SA [39,40]. If the basal level of endogenous SA in wt plants at least partially inhibits HMGB3’s DAMP activity, we would expect HMGB3-induced MAPK activation and callose deposition to be further enhanced in *sid2* compared to wt plants. In contrast, extracellular HMGB3-induced MAPK activation and callose deposition levels were similar in *sid2* and wt plants (**S6 Fig**), suggesting that basal levels of endogenous SA are insufficient to suppress HMGB3’s DAMP activity.

**Fig 6:**
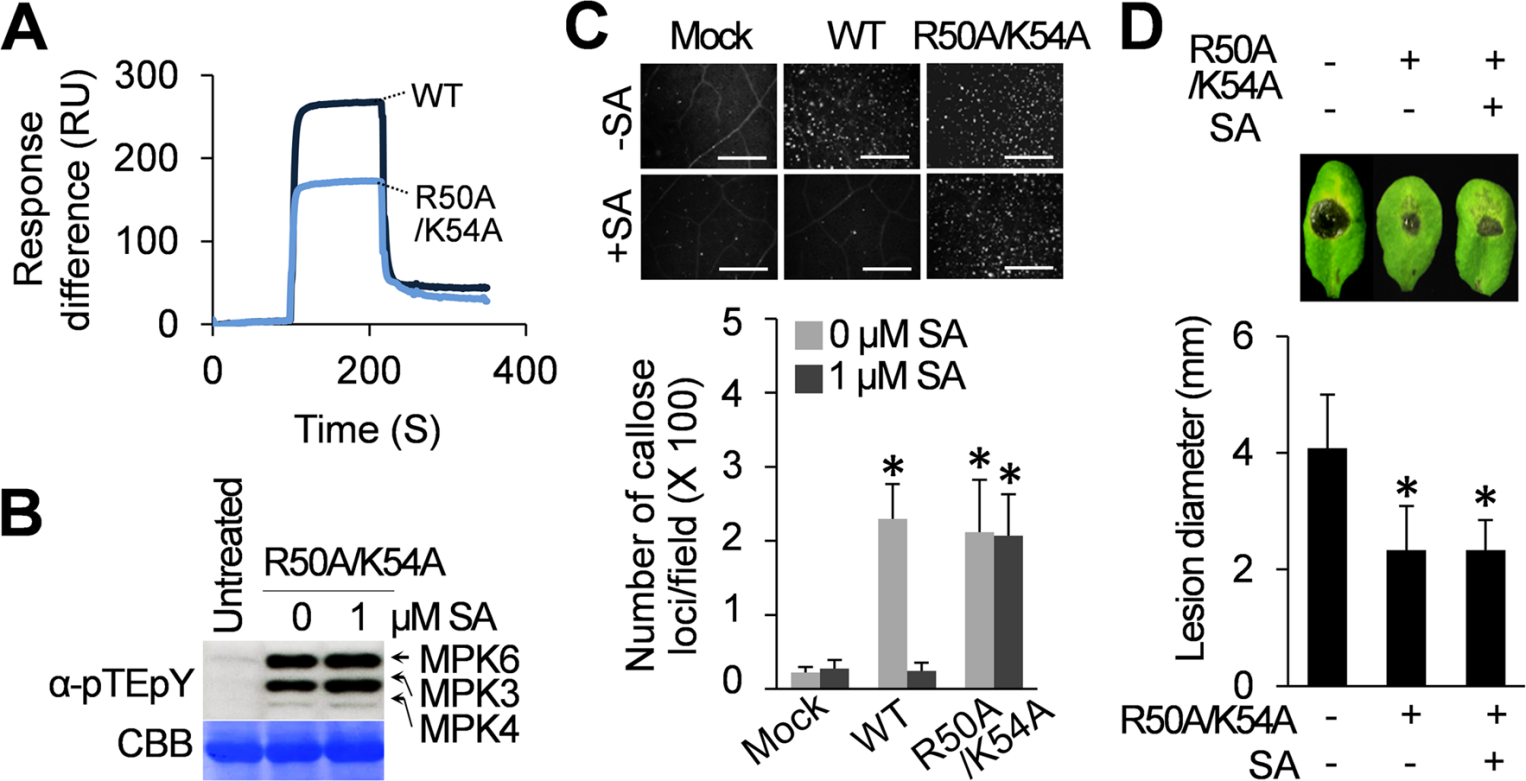
Arg50 and Lys54 are required for SA to bind and inhibit HMGB3’s DAMP activity. **A.** Sensorgrams obtained with 1 μM wild type (WT) HMGB3 and the R50A/K54A mutant using a 3AESA-immobilized sensor chip. The signals detected from a mock-coupled control chip were subtracted. **B.** The ability of the R50A/K54A mutant to activate MAPKs was not suppressed by SA. Leaves were sampled 15 min after infiltration with water containing 1 μM R50A/K54A with or without 1 μM SA. Phosphorylated MPK3, MPK4, and MPK6 were detected by α-pTEpY antibody. Rubisco large subunit protein stained with CBB served as the loading control. **C.** R50A/K54A-induced callose deposition was not suppressed by SA. Leaves were sampled 15 h after infiltration with water containing 0.1 μM HMGB3 (WT) or 0.1 μM R50A/K54A with or without 1 μM SA. Representative pictures for callose staining are shown in the upper panel. Bars = 100 μm. Data are the mean ± SD (lower panel, n = 20). Leaves infiltrated with water served as mock control. Asterisks indicate a significant difference from the mock-treated leaves (*t* test, *P* < 0.05). **D.** SA failed to inhibit the enhanced resistance to *B*. *cinerea* induced by mutant HMGB3 (R50A/K54A). Leaves were infiltrated with water containing 0.1 μM mutant HMGB3 with or without 1 μM SA one day before *B*. *cinerea* infection. Representative disease symptoms at 3 dpi are shown in the upper panel. Data corresponding to this time point are presented as the mean ± SD (lower panel, n = 6). Asterisks indicate a significant difference from the mock-treated leaves (*t* test, *P* < 0.05). Leaves infiltrated with water served as a control.

In comparison to hHMGB1, which contains two HMG boxes designated Box A and Box B, Arabidopsis HMGBs contain only one HMG box. Comparison of the sequences among these HMG box domains revealed that the critical SA-binding residues of hHMGB1, Arg50 and Lys54, are conserved in Arabidopsis HMGBs 1,2,3,6 and 12 (**S7 Fig**). Thus, these residues were mutated to Ala, and the resulting HMGB3 R50A/K54A mutant was tested for SA binding activity, as well as its ability to induce immune responses in the absence or presence of SA. SPR analysis revealed that the R50A/K54A mutant has reduced binding to the 3AESA-immobilized chip compared with WT HMGB3 (**Fig 6A**). Importantly, the R50A/K54A HMGB3 mutant retained its ability to activate MPK3 and MPK6, induce callose deposition, and enhance resistance, but these activities were no longer inhibited by co-infiltrated SA (**Fig 6B, 6C** and **6D**).

## Discussion

Here we report that HMGB3 functions as a DAMP *in planta*. It is released into apoplast upon necrotrophic pathogen infection and induces innate immune responses including: i) MAPK activation ii) expression of defense-related genes iii) callose deposition, and iv) enhanced resistance to pathogen infection. Although the receptor for HMGB3 has yet to be identified, we demonstrate that HMGB3-mediated induction of MAPK activation, callose deposition, and enhanced resistance to *B*. *cinerea* requires the LRR-RLKs BAK1 and BKK1, like the well-characterized DAMP Pep1. Interestingly, in contrast to ProPep1, whose expression was moderately induced by treatment with a prototypic MAMP flg22, the 22 amino acid active epitope of flagellin, this treatment did not enhance HMGB3 expression (**S8 Fig**). This is perhaps not surprising since HMGBs are among the most abundant proteins in the cell and therefore, their levels release into the apoplast during necrosis are likely to be sufficiently high to induce immune responses. Consistent with our results, expression of the recently identified human DAMP peroxiredoxin 2, also was not induced by bacterial lipopolysaccharides [41]. Together these results demonstrate that the expression of a DAMP-encoding gene is not necessarily induced by MAMPs.

**Fig 7:**
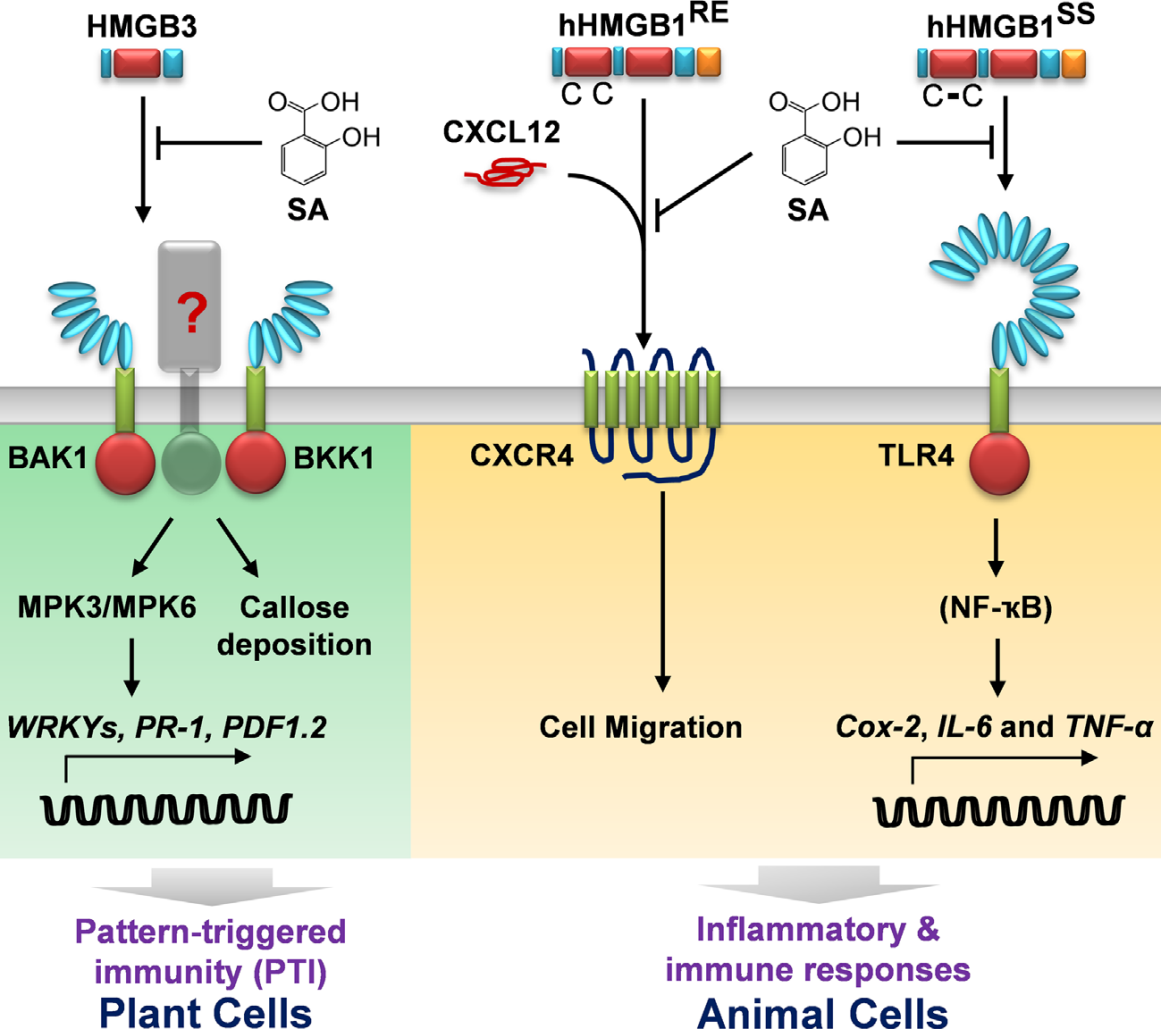
Schematic of the mechanisms through which SA inhibits the DAMP activities of HMGBs in plant and animal cells. In plant cells, extracellular HMGB3 is recognized by a yet to be identified receptor complexed with the regulatory LRR RLKs BAK1 and/or BKK1. Recognition induces a series of pattern-triggered immune (PTI) responses, including MAPK activation (MPK3 and MPK6), defense-related gene expression (WRKYs, *PR-1*, and *PDF1*.*2*), and callose deposition. SA binding to extracellular HMGB3 inhibits its DAMP activity. In animal cells, extracellular HMGB1 is recognized by multiple cell surface receptors, including C-X-C chemokine receptor 4 (CXCR4) and toll-like receptor 4 (TLR4), depending on its redox state. SA inhibits chemo-attractant activity of reduced HMGB1 (hHMGB1^RE^), which is recognized by the cell surface receptor CXCR4 after heterocomplex formation of hHMGB1^RE^ with C-X-C motif-containing chemokine 12 (CXCL12) [24,42]. SA also inhibits activation of *Cox-2* and pro-inflammatory cytokine genes (*IL-6* and *TNF-*α) by the disulfide-bonded form of HMGB1 (hHMGB1^SS^), which is mediated by heterocomplex formation of hHMGB1^SS^ with myeloid differentiation factor 2 (MD-2), followed by signal transduction through the cell surface receptor TLR4 and transcription factor NF-κB [23,24].

Recently, we reported that aspirin’s primary metabolite in humans SA interacts with HMG box of hHMGB1, thereby inhibiting the pro-inflammatory activities of extracellular hHMGB1 [24] (**Fig 7**). Binding of SA by fully-reduced HMGB1 (hHMGB1^RE^) inhibits its chemo-attractant activity, which stimulates migration of immune cells to the site of tissue damage. SA also suppresses induction by the disulfide-bonded form of HMGB1 (hHMGB1^SS^) through the TLR4 receptor of expression of pro-inflammatory cytokine genes, such as *IL-6* and *TNF-α*, as well as *Cox-2*. Here we demonstrated that Arabidopsis HMGB3, like hHMGB1, binds SA, which inhibits its, but not Pep1’s, DAMP activity *in planta*. Thus, SA-mediated suppression of HMGB3-induced immune responses appears to be due to its specific interaction with HMGB3, rather than a general suppression of DAMP signaling. Supporting this hypothesis, mutations in HMGB3’s HMG box, which correspond to the SA-binding sites identified in hHMGB1, did not affect HMGB3’s DAMP activity, but suppressed SA binding and the resulting inhibition by SA of its DAMP activity.

HMGBs are present in all eukaryotes, where they function as very abundant non-histone nuclear proteins that help to maintain and regulate chromatin structure. In addition, mammalian HMGB1 functions as a DAMP following its passive release from damaged or dying cells or its active secretion into the extracellular milieu [17,43]. hHMGB1 does not have a typical hydrophobic secretory signal peptide, but possesses two separate nuclear localization signals (NLS1: amino acids 28–44 and NLS2: amino acids 179–185) [17]. Acetylation and/or phosphorylation of the NLS redirects hHMGB1 toward active secretion in immune cells, such as monocytes and macrophages and in severely stressed cells such as certain cancer cells [17,44–47]. In contrast, plants do not have specialized immune cells, and the post-translational modifications that lead to active secretion of hHMGBs from the nucleus into the cytoplasm or extracellular space have not been reported in plants. In Arabidopsis HMGBs, specific NLS have not been identified; rather it appears that a basic region in their N-termini mediate nuclear translocation, at least for HMGB1/5, which are exclusively localized in the nucleus [26]. For HMGB2/3/4, their nucleo-cytoplasmic distribution depends on the balance between the basic N-terminal and the acidic C-terminal regions flanking the central HMG box [27]. Interestingly, serine residues in the C-terminal acidic tail of HMGBs from maize, broccoli, and Arabidopsis can be phosphorylated by protein kinase CK2α [48–50]. However, the phosphorylation status does not alter the nucleo-cytoplasmic distribution of Arabidopsis HMGB2, but rather modulated its intra-nuclear distribution [27,49].

At present there is no evidence for active secretion of HMGB3. Rather it likely gains access to the extracellular space passively i) when cells are damaged mechanically, or by herbivores including insects, or ii) during infection by necrotrophic pathogens. Indeed, we observed that infection by necrotrophic *B*. *cinerea* caused release of HMGB3 into the apoplast within 24 hours after inoculation. Such release into the apoplast during the early phase of cellular necrosis induced by necrotrophs could enhance resistance by inducing immune responses. In contrast, release of HMGB3 into the apoplast by biotrophic pathogens is less probable. Thus, HMGB3-induced immune responses may have evolved to protect against necrotrophs and insects, rather than against biotrophs or against hemi-biotrophs, which induce necrosis only at a late stage of infection, probably too late for the immune responses induced by its released HMGB3 to significantly affect the outcome of the infection. Consistent with this hypothesis, infiltration of HMGB3 into the apoplast failed to protect against the hemi-biotrophic pathogen *Pseudomonas syringae* pv. *tomato* (**S9 Fig**).

SA has been shown to play a central role in plant disease resistance. It acts at several levels of immunity through multiple SA-binding proteins to reprogram transcription [36,37,51,52], modulate cellular redox [53,54], inhibit the activity of a host factor usurped by a virus for its replication [38], etc. However, while SA-induced defense responses are critical for resistance to biotrophic and hemi-biotrophic pathogens, the main regulator for defense against necrotrophic pathogens and insects is jasmonic acid (JA) [55,56]. Given that the JA and SA defense signaling pathways are often mutually antagonistic, SA-mediated inhibition of HMGB3 may provide a mechanism for this cross-talk. In this scenario, release of HMGB3 to the extracellular space would occur when cells are damaged during infection by necrotrophic pathogens. Once in the apoplast, HMGB3’s DAMP activity would activate JA/ethylene-associated defenses to help neutralize these threats. Interestingly, the similar induction of immune responses by HMGB3 in wt and the SA-deficient *sid2* mutant suggest that the basal level of SA in uninfected plants, at least in their apoplast, is too low to effectively inhibit HMGB3’s DAMP activity. By contrast, infection with a biotrophic pathogen leads to an increase in SA levels [55,56]. These elevated SA levels could antagonize the activation of JA-associated defenses by suppressing HMGB3’s DAMP activity, while promoting the activation of SA-associated defenses that are more effective against this type of pathogen.

In summary, the identification of extracellular HMGB3 as a novel plant DAMP whose immune response-inducing activity is inhibited by SA binding provides cross-kingdom evidence that HMGB proteins function extracellularly as DAMPs in both plants and animals. It also highlights the presence of common targets and shared mechanisms of action for SA in plants and humans.

## Materials and Methods

### Expression and purification of HMGB3

The ORF clones for Arabidopsis HMGB3 were obtained from Arabidopsis Biological Resource Center (ABRC). HMGB3 was amplified and cloned into *Kpn*I and *Eco*RI sites of pET30 Xa/LIC. To generate the mutant HMGB3 (R50A/K58A) expression clone, a series of PCR-based point mutations was generated by using the oligonucleotides listed in **S1 Table**. Maltose binding protein (MBP) is expressed by using pET-MALHT vector [37]. Recombinant MBP and HMGB3 proteins, including the mutant HMGB3, were expressed in *Escherichia coli* strain BL21 cells grown at 37°C to OD_600_ = 0.7. Expression was induced by adding 0.1 mM isopropyl-β-D-thiogalactopyrandoside (IPTG) for 16 h at room temperature. Cells were collected by centrifugation at 6,000 *g* for 30 min and stored at -20°C. For protein purification, cells were resuspended in lysis buffer, buffer A (20 mM Tris-HCl/8.0, 0.15 M NaCl and 10% glycerol) plus 0.2% NP-40, 1 mg/ml lysozyme, 1 mM phenylmethyl sulphonyl fluoride, and 10 mM imidazole. After sonication and centrifugation at 50,000 g for 1 h, soluble 6×His-tagged MBP and HMGB3 was purified by affinity chromatography using Ni-NTA agarose resin (Novagen) as follows. After running the protein extract onto the column the MBP- or HMGB3-bound resin was washed with buffer A containing 20 mM imidazole and 0.5% Triton X-114 to remove *Escherichia coli*-derived endotoxin, including lipopolysaccharide [57]. Column-bound MBP or HMGB3 was eluted in buffer A containing 300 mM imidazole. MBP and HMGB3 was further purified using gel filtration chromatography on a HiLoad 16/60 Superdex 200 column (GE Healthcare) equilibrated in buffer A. Each preparation of purified protein was tested for endotoxin levels using the Toxinsensor kit (Genscript, USA). Purified MBP and HMGB3 containing less than 50 pg endotoxin per 1 mg of HMGB3 protein were used for infiltration experiments.

### MAPK activation assay

To detect the phosphorylation of Arabidopsis MAPKs (MPK3, MPK4, and MPK6), 4 week-old soil grown Arabidopsis leaves were infiltrated with water containing either 1 μM recombinant HMGB3 protein or 1 μM Pep1 peptide (ATKVKAKQRGKEKVSSGRPGQHN; synthesized by GenScript USA Inc.). Leaves infiltrated with water served as mock control. Two leaf disks were cut from the leaf center of two different leaves using the cork borer (diameter = 0.7 cm), and immediately frozen and ground to fine powder in liquid nitrogen. Proteins were extracted in lacus buffer (25 mM Tris–HCl, pH 7.5, 15 mM MgCl_2_, 15 mM EGTA, 75 mM NaCl, 1 mM DTT, 0.1% NP-40, 5 mM p-nitrophenylphosphate, 60 mM β-glycerophosphate, 0.1 mM Na_3_VO_3_, 1 mM NaF, 1 mM PMSF, 5μg/ml leupeptin, 5 μg/ml aprotinin), and then centrifuged at 15,000 *g* for 10 min at 4°C to remove the cell debris. Protein concentration of supernatant is measured using the Bradford reagent (Bio-Rad). Forty μg of proteins per sample was separated on the 8% SDS-PAGE and transferred onto the PVDF membrane for immunoblotting (IB) using the anti-phospho-p44/42 MAPK antibody (Cell Signaling Technology). The large subunit of ribulose-1,5-bisphosphate carboxylase/oxygenase (Rubisco) was visualized by Coomassie Brilliant Blue (CBB) staining of PVDF membrane that was used for IB to assess consistency of loading. For quantification of relative MAPK activation in the three genetic backgrounds densitometry was performed with Image J software. First, the band intensities of the phosphorylated MAPKs were normalized to Rubisco large subunit stained with CBB. The normalized levels in the two mutant plants were then compared to that in wt plants to determine the percentage reduction reported in the text. Experiments were done at least two times with similar results.

### Induction and analyses of gene expression

The expression of *HMGBs* and defense-related maker genes were examined using the real-time RT-PCR (qRT-PCR) technique. To analyze the gene expression by extracellular HMGB3 or Pep1, 4-week-old soil grown Arabidopsis leaves were infiltrated with water containing either 1 μM HMGB3 or Pep1 peptide, unless otherwise indicated. Treatment with water served as mock control. Total RNA was extracted using TRIzol Reagent (Invitrogen), followed by DNase treatment (Ambion) according to the manufacturer's instructions. The first-strand cDNA was synthesized from the total RNA (~2 μg) with Superscript III reverse transcriptase (Invitrogen) and oligo (dT)_15_ primer (Integrated DNA Technologies). Two μL of 20 times diluted cDNAs were used for quantitative real-time RT PCR (qRT-PCR) with IQ SYBR Green Supermix (Bio-Rad) and gene-specific primer pairs as listed in **S2 Table**. Amount of expression of the genes in each sample was normalized by using the expression levels of *tubulin beta-4* (*TUB4*) as an internal control, and then the relative expression level was calculated by using the ΔΔdCt analyses. Experiments were done at least two times with similar results.

### Callose staining

Arabidopsis leaves collected 15 h after infiltration with water containing 0.1 μM HMGB3 or 0.1 μM Pep1, were immediately immersed in alcoholic lactophenol (phenol:glycerol:lactic acid:water:ethanol = 1:1:1:1:8) to destain the chlorophyll. To remove the background fluorescence, destained leaves are stained with toluidine blue O solution (0.05% toluidine blue O, 0.1 M sodium acetate, pH 4.4) for 30 min, and then rinsed twice with 150 mM K_2_HPO_4_ (pH 9.5). Callose was stained with aniline blue solution (0.02% aniline blue, 150 mM K_2_HPO_4_, pH 9.5) for 30 min under the dark condition. Aniline blue-stained leaves were mounted with 10% glycerol and observed using a Leica DM5500 Epifluorescence Microscope (Plant Cell Imaging Center, Boyce Thompson Institute for Plant Research). The number of callose loci was quantified using the ImageJ (NIH). Experiments were done at least two times with similar results.

### *B*. *cinerea* infection

*B*. *cinerea* B.05.10 was grown on 2×V8 agar media (36% V8 juice, 0.2% CaCO_3_, and 2% Bacto-agar). Fungal mycelia and spores were collected by scraping the surface with 1% *Sabouraud* maltose broth (Difco), and then vigorously vortexed to release the spores. The suspension was passed through Miracloth to separate the fungal spores from mycelia and pieces of agar. To infect the plants, the concentration of spore were adjusted to 10^4^ spores/mL. Five-μL of spore suspension was dropped on the adaxial surface of fully expanded Arabidopsis leaves. The inoculated plants were kept under a transparent cover to maintain high humidity. Experiments were done at least two times with similar results.

### Generation of *amiR*-*hmgbs* transgenic Arabidopsis plants

To generate the *HMGB3*-silencing constructs, an artificial microRNA (amiRNA) was constructed based on prediction by WMD3-Web MicroRNA designer (http://wmd3.weigelworld.org/) [58,59]. Among the various predicted amiRNAs, an *amiR*-*hmgbs* (5’-TAAGAAGGCACTGGGAGGCCT-3’) was selected based on its possible multi-target silencing activity, including *HMGB1*, *HMGB2* and *HMGB3* (**S4 Fig**). The *amiR*-*hmgbs* was introduced into pRS300, which contains the miR319a precursor in pBSK, by using the primers listed in **S1 Table**, and then cloned into the pBTEX vector via the *Eco*RI/*Bam*HI restriction enzyme sites to yield pBTEX:*amiR-hmgbs*. This clone was transformed into the *Agrobacterium tumefaciens* strain GV3101 and used for Arabidopsis floral dip transformation. Transgenic Arabidopsis plants (T_1_) harboring the transgenes were selected by planting the seeds on Murashige and Skoog plates containing 25 mg/L kanamycin and 50 mg/L timentin. The T_2_ generations of transgenic lines were used for further experiments.

### Localization of HMGB3

The ORF region for Arabidopsis *HMGB3* was amplified using the gene-specific primers listed in **S1 Table** and cloned into pMD1 binary vector [60] using *Sal*I/*Sac*I restriction enzyme sites to yield pMD1:*HA*:*HMGB3*. *A*. *tumefaciens* GV3101 containing empty vector (pMD1:HA) or pMD1:HA:HMGB3 construct was grown overnight at 28°C on an LB agar plate containing 50 mg/L kanamycin, 50 mg/L gentamycin and 50 mg/L rifampicin. Bacterial cells were washed two times and resuspended in infiltration buffer [10 mM 4-morpholineethanesulfonic acid (MES, pH 5.7), 10 mM MgCl_2_ and 200 μM acetosyringone] to a final OD_600_ of 0.4. Bacterial suspensions were infiltrated into *Nicotiana benthamiana* leaves using a needless syringe. Expression of HMGB3 was detected from the leaves 2 d after agro-infiltration by IB with α-HA antibody (Sigma-Aldrich). For *B*. *cinerea* infection assay, the concentration of spores was adjusted to 10^5^ spores/mL in 1% *Sabouraud* maltose broth (Difco) with 0.03% tween 20, and used for spray inoculation 1 d after agro-infiltration. Leaves were detached and incubated on a water agar plate to maintain the humidity. Subcellular protein fractionation was performed as described previously [61–63]. Briefly, the leaves expressing empty vector or HMGB3 were vacuum-infiltrated with apoplastic washing fluid (AWF) extraction buffer (10 mM phosphate, 0.1% sodium metabisulphite, pH 6.8), and then the outsides of the leaves were washed three times with sterile water at 4°C. Leaves were gently dried with a paper towel and then centrifuged at 1,000 x g for 10 min in a swing bucket rotor at 4°C to collect the AWF. The collected AWF was centrifuged at 15,000 x g for 5 min to remove any cells or particular matter. Proteins in the AWF were precipitation with a 4-fold excess (v/v) of cold acetone. Leaf tissue used for AWF collection were stored at -80°C before preparation of total leaf extracts or subcellular fractions. Total leaf extracts were prepared by grinding 2 g of leaf tissues in 4 ml of lysis buffer [20 mM Tris-HCl (pH 7.4), 25% glycerol, 20 mM KCl, 2 mM EDTA, 2.5 mM MgCl_2_, 250 mM sucrose, 1 mM dithiothreitol (DTT), and complete protease inhibitor cocktail (Roche)] and then sequentially filtered through the 100 μm and 40 μm nylon meshes (Falcon). The filtered homogenate were centrifuged at 1,500 x g at 4°C for 10 min to pellet the nuclei. Supernatant served as cytosolic fraction. Nuclear pellets were washed three times with nuclei resuspension buffer [20 mM Tris-HCl (pH 7.4), 25% glycerol and 2.5 mM MgCl_2_] with 0.2% Triton X-100, and then resuspended in nuclei resuspension buffer. Ten μg of protein from the AWF, total leaf extract, or subcellular fractions were used for IB with α-HA antibody (Sigma-Aldrich). Histone H3 level were monitored by IB with α-histone H3 (Abcam) with all four fractions to monitor nuclear contamination or release of hostone H3 intio the apoplast during *B*. *cinerea* infection.

### Assessment of SA-binding activity of HMGB3

SA-binding activity was assessed by photo-affinity labeling and SPR as previously described [36,37,64]. Briefly, purified His-tagged recombinant proteins (2 μg) were incubated 1 h on ice with 4AzSA (50 μM) in 40 μl 1X PBS without or with various concentrations of excess SA, followed by UV irradiation with 254 nm UV light at an energy level of 30 mJ using a GS GENE linker™ UV chamber (Bio-Rad). Ten μl of reaction mixture were subjected to SDS-PAGE followed by immuno-blotting with α-SA antibody (Acris) to detect 4AzSA-crosslinked proteins. For SPR experiments, 3AESA was immobilized on the CM5 sensor chip as described previously [36]. To test SA binding of HMGB3, proteins were filtered and diluted in HBS-EP buffer with or without various concentrations of SA, and then flowed over the sensor chip with 3AESA immobilized or over the mock-coupled sensor chip. The binding signal was determined by subtracting the signal from the mock-coupled chip from the signal from the 3AESA immobilized chip. The sensor chips were regenerated by stripping off bound protein with NaOH solution (pH12). Experiments were done at least two times with similar results.

### Statistical analysis

The results are expressed as a mean ± standard deviation (SD). Statistical analyses for statistical significance between different means was determined using a *t* test (P<0.05).

## Supporting Information

S1 Fig Extracellular HMGB3 specifically induces MAPK activation and callose deposition in Arabidopsis. **A.** MAPK activation in Arabidopsis. Leaves were collected 15 min after infiltration with water containing either 20 μg/mL HMGB3 (= 1 μM) or 20 μg/mL MBP for the MAPK activation assay. Activation of MPK3, MPK4, and MPK6 by MAPK kinase-mediated phosphorylation of the TEY sequence was detected with α-pTEpY antibody. Rubisco large subunit protein stained with CBB served as a loading control. **B.** Callose deposition in Arabidopsis. Leaves were stained with aniline blue 15 h after infiltration with water containing either 2 μg/mL HMGB3 (= 100 nM) or 2 μg/mL and 20 μg/mL (10X) MBP. Representative pictures are shown in the upper panel. Bars = 100 μm. Data are the mean ± SD (right panel, n = 20). The asterisk indicates significant difference from mock-treated plants.
(TIF)

S2 Fig Mutant and wild-type HMGB3 confer similar resistance to *B*. *cinerea*. Fungal biomass determined by qRT-PCR 3 d after *B*. *cinerea* infection in wild-type Col-0 plants. Leaves were infiltrated with water (Mock) or water containing 0.1 μM wild-type (WT) or mutant (R50A/K54A) HMGB3 one day before *B*. *cinerea* infection. *B*. *cinerea Actin* genomic DNA levels are shown relative to the level of the *Arabidopsis Actin* [32]. Mock-treated sample is set to 100% relative quantity. Asterisks indicate a significant difference from the mock-treated leaves (t test, P < 0.05).
(TIF)

S3 Fig HMGB3-induced defense-related gene expression is compromised in *bak1-5/bkk1-1*. Four-week-old wt or *bak1-5*/*bkk1-1* mutant Arabidopsis leaves were collected 30 min after infiltration with water containing either 0 μM or 1 μM HMGB3. Following RT-PCR, expression levels were plotted relative to the expression in water-treated wt leaves (mock), which was set at 1. Data are the mean ± SD (n = 4). Asterisks indicate significant differences between HMGB3- and mock-treated leaves while # indicates significant difference between responses of wt and *bak1*/*bkk1* plants (*t* test, *P* < 0.05).
(TIF)

S4 Fig Predicted *HMGB* targets of *amiR-hmgbs*. **(A)** To generate the *HMGB3*-silencing constructs, an artificial microRNA (amiRNA) was constructed based on a prediction by WMD3-Web MicroRNA designer (http://wmd3.weigelworld.org/) [58,59]. Among the various predicted amiRNAs, an *amiR*-*hmgbs* (5’-TAAGAAGGCACTGGGAGGCCT-3’) was selected based on its possible multi-target silencing activity, including *HMGB1*, *HMGB2*, and *HMGB3*. **(B)** Nucleotide sequence alignment of Arabidopsis *HMGB*s with *amiR*-*hmgbs*. Multiple sequence alignments between Arabidopsis *HMGB*s and *amiR-hmgbs* were performed using the Clustal Omega program (Version 1.2.1) [65]. The consensus nucleotide (nt) sequences are presented in white with a black background. The number of identical nucleotides in the sequences of *HMGB*s and *amiR-hmgbs* are shown in parentheses in red. *HMGB1* (AT3G51880), *HMGB2* (AT1G20693), *HMGB3* (AT1G20696), *HMGB4* (AT2G17560), *HMGB5* (AT4G35570), *HMGB6* (AT5G23420), *HMGB12* (AT5G23405) and *HMGB14* (AT2G34450).
(TIF)

S5 Fig Defense-related gene expression in uninfected *amiR*-*hmgbs* transgenic lines. There was no significant difference in the expression of *PDF1*.*2* or *PR-1* in untreated wt plants and the *amiR-hmgbs* transgenic line #3. Expression levels determined by RT-PCR were plotted relative to the expression in untransformed wt leaves, which were set at 1. Data are the mean ± SD (n = 4).
(TIF)

S6 Fig Basal endogenous level of SA does not affect HMGB3’s DAMP activity. **A.** MAPK activation in the SA-deficient *sid2* mutant Arabidopsis. Leaves were collected 15 min after infiltration with water containing 1 μM HMGB3 for the MAPK activation assay. Activation of MPK3, MPK4, and MPK6 by MAPK kinase-mediated phosphorylation of the TEY sequence was detected with α-pTEpY antibody. Rubisco large subunit protein stained with CBB served as a loading control. **B.** Callose deposition in Arabidopsis. Leaves were stained with aniline blue 15 h after infiltration with water containing 0.1 μM HMGB3 (= 100 nM) or 2 μg/mL and 20 μg/mL (10X) MBP. Representative pictures are shown in the upper panel. Bars = 100 μm. Data are the mean ± SD (right panel, n = 20).
(TIF)

S7 Fig Amino acid sequence alignment of human and plant high mobility group (HMG) boxes. Amino acid sequence alignment of HMG boxes from human and Arabidopsis HMGBs. Human HMGB1 (CAG33144) contains two HMG boxes, designated Box A and Box B [24], whereas the Arabidopsis HMGBs contains only one HMG box: HMGB1 (AT3G51880), HMGB2 (AT1G20693), HMGB3 (AT1G20696), HMGB4 (AT2G17560), HMGB5 (AT4G35570), HMGB6 (AT5G23420), HMGB12 (AT5G23405) and HMGB14 (AT2G34450). Multiple sequence alignments were performed using the Clustal Omega program (Version 1.2.1) [65]. Conserved Arg (R) and Lys (K) residues, which are critical for SA binding, are highlighted in yellow and denoted with yellow asterisks [24]. The consensus and similar amino acid sequences are highlighted by black and grey backgrounds, respectively. Dashes indicate spacing in the amino acid sequences required for proper alignment.
(TIF)

S8 Fig Quantitative RT-PCR analyses of HMGB3 and PROPEP1 expression 1h after flg22 treatment. Arabidopsis leaves were infiltrated with water without (Mock) or with 1 μM flg22. Data are the mean ± SD (n = 3). The asterisk indicates significant difference between flg22- and mock-treated leaves (t test, P < 0.05).
(TIF)

S9 Fig Extracellular HMGB3 did not induce resistance to hemi-biotrophic pathogen *Pseudomonas syringae*. Leaves were infiltrated with the indicated concentrations of HMGB3 one day before *P*. *syringae* pv. *tomato* (*Pst*) DC3000 inoculation (10^5^ cfu/ml). Bacterial growth was examined 2 days after inoculation. Data are the mean ± SD (n = 5). There were no significant differences between HMGB3- and mock-treated leaves (*t* test, *P* < 0.05).
(TIF)

S1 Table Oligonucleotides used for cloning in this study. (DOCX)

S2 Table Oligonucleotides used for real-time qPCR in this study. (DOCX)
