# Supplementary figures and images for: Activation of Plant Innate Immunity by Extracellular High Mobility Group Box 3 and Its Inhibition by Salicylic Acid
Source: PLoS Pathog. 2016 Mar 23;12(3):e1005518. doi: 10.1371/journal.ppat.1005518 (PMC4805298; doi:10.1371/journal.ppat.1005518)

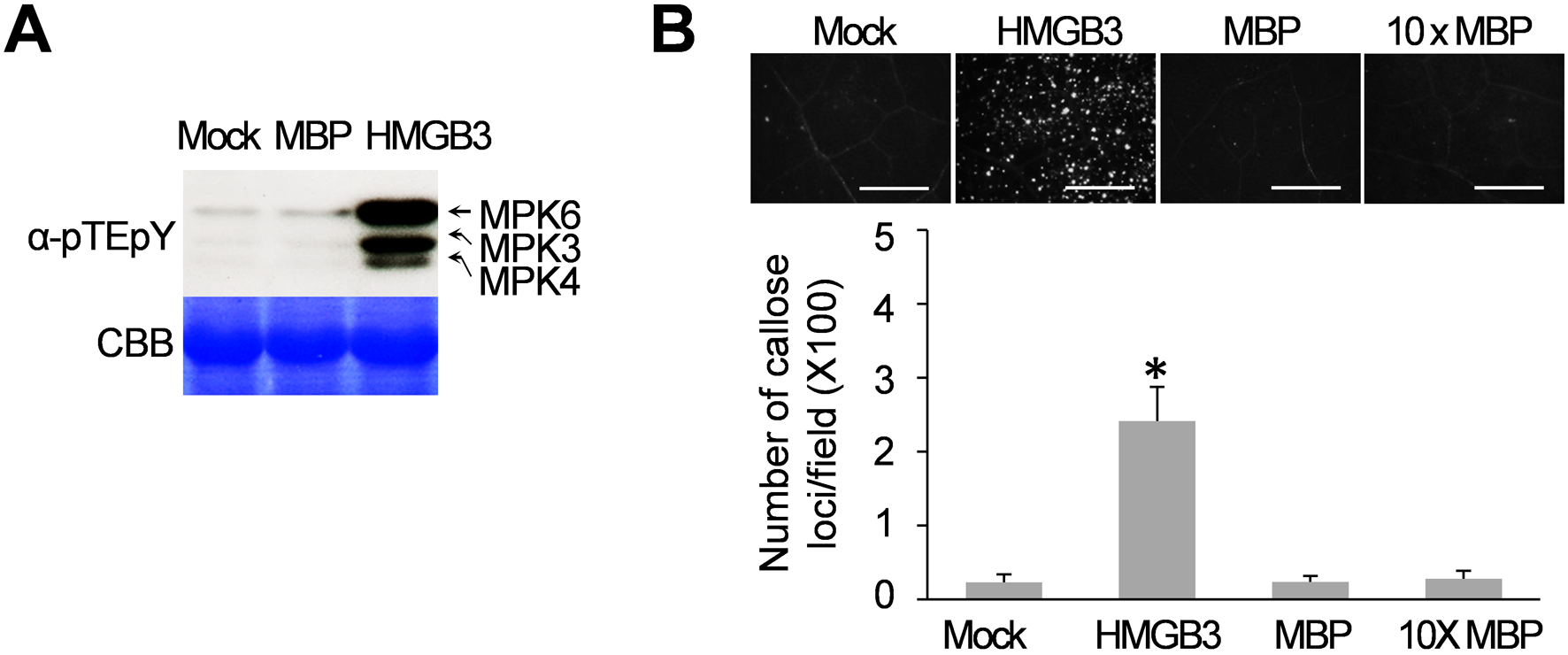

Supplement: S1 Fig — A. MAPK activation in Arabidopsis. Leaves were collected 15 min after infiltration with water containing either 20 μg/mL HMGB3 (= 1 μM) or 20 μg/mL MBP for the MAPK activation assay. Activation of MPK3, MPK4, and MPK6 by MAPK kinase-mediated phosphorylation of the TEY sequence was detected with α-pTEpY antibody. Rubisco large subunit protein stained with CBB served as a loading control. B. Callose deposition in Arabidopsis. Leaves were stained with aniline blue 15 h after infiltration with water containing either 2 μg/mL HMGB3 (= 100 nM) or 2 μg/mL and 20 μg/mL (10X) MBP. Representative pictures are shown in the upper panel. Bars = 100 μm. Data are the mean ± SD (right panel, n = 20). The asterisk indicates significant difference from mock-treated plants. (TIF) [file ppat.1005518.s001.tif]

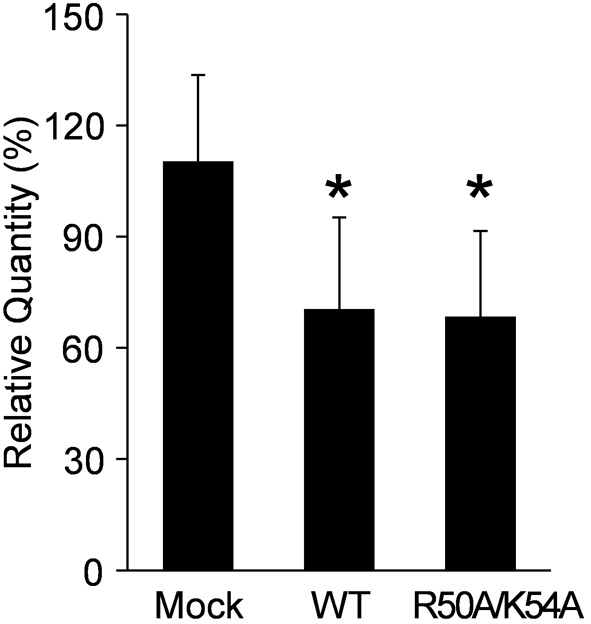

Supplement: S2 Fig — Fungal biomass determined by qRT-PCR 3 d after B. cinerea infection in wild-type Col-0 plants. Leaves were infiltrated with water (Mock) or water containing 0.1 μM wild-type (WT) or mutant (R50A/K54A) HMGB3 one day before B. cinerea infection. B. cinerea Actin genomic DNA levels are shown relative to the level of the Arabidopsis Actin [32]. Mock-treated sample is set to 100% relative quantity. Asterisks indicate a significant difference from the mock-treated leaves (t test, P < 0.05). (TIF) [file ppat.1005518.s002.tif]

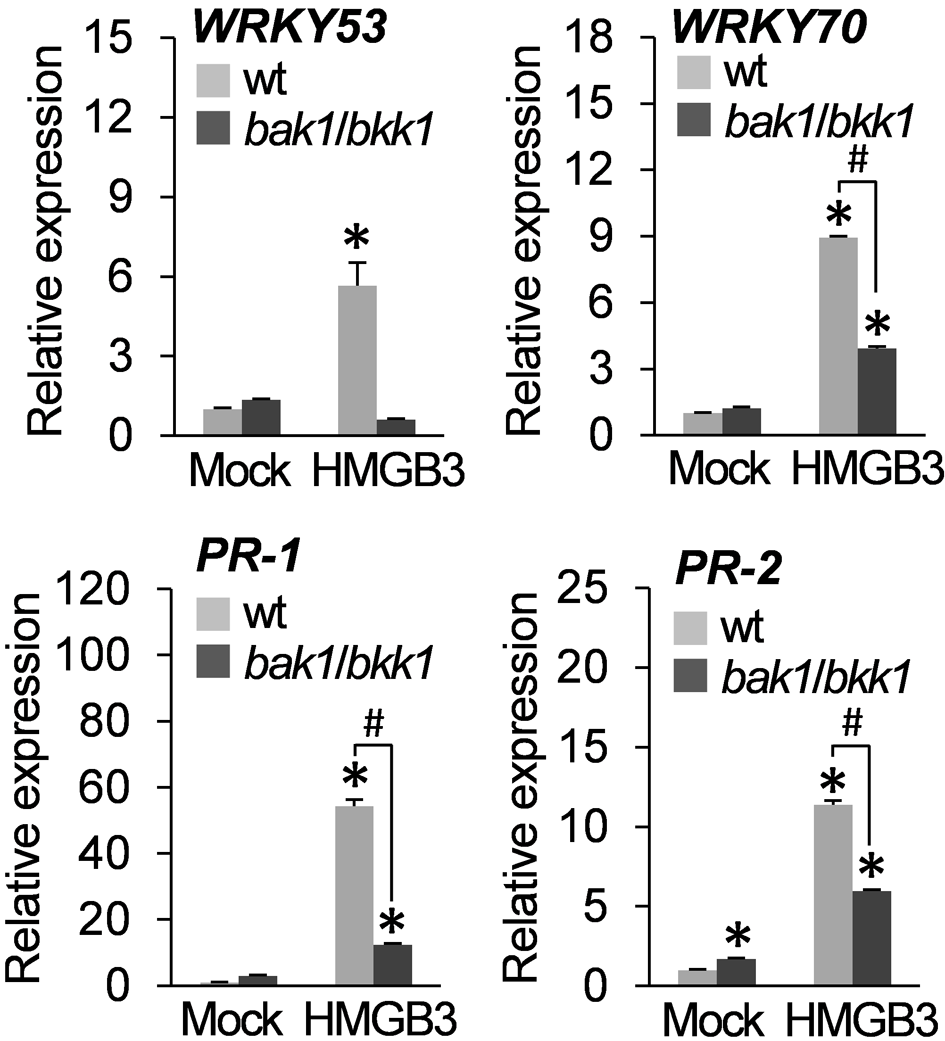

Supplement: S3 Fig — Four-week-old wt or bak1-5/bkk1-1 mutant Arabidopsis leaves were collected 30 min after infiltration with water containing either 0 μM or 1 μM HMGB3. Following RT-PCR, expression levels were plotted relative to the expression in water-treated wt leaves (mock), which was set at 1. Data are the mean ± SD (n = 4). Asterisks indicate significant differences between HMGB3- and mock-treated leaves while # indicates significant difference between responses of wt and bak1/bkk1 plants (t test, P < 0.05). (TIF) [file ppat.1005518.s003.tif]

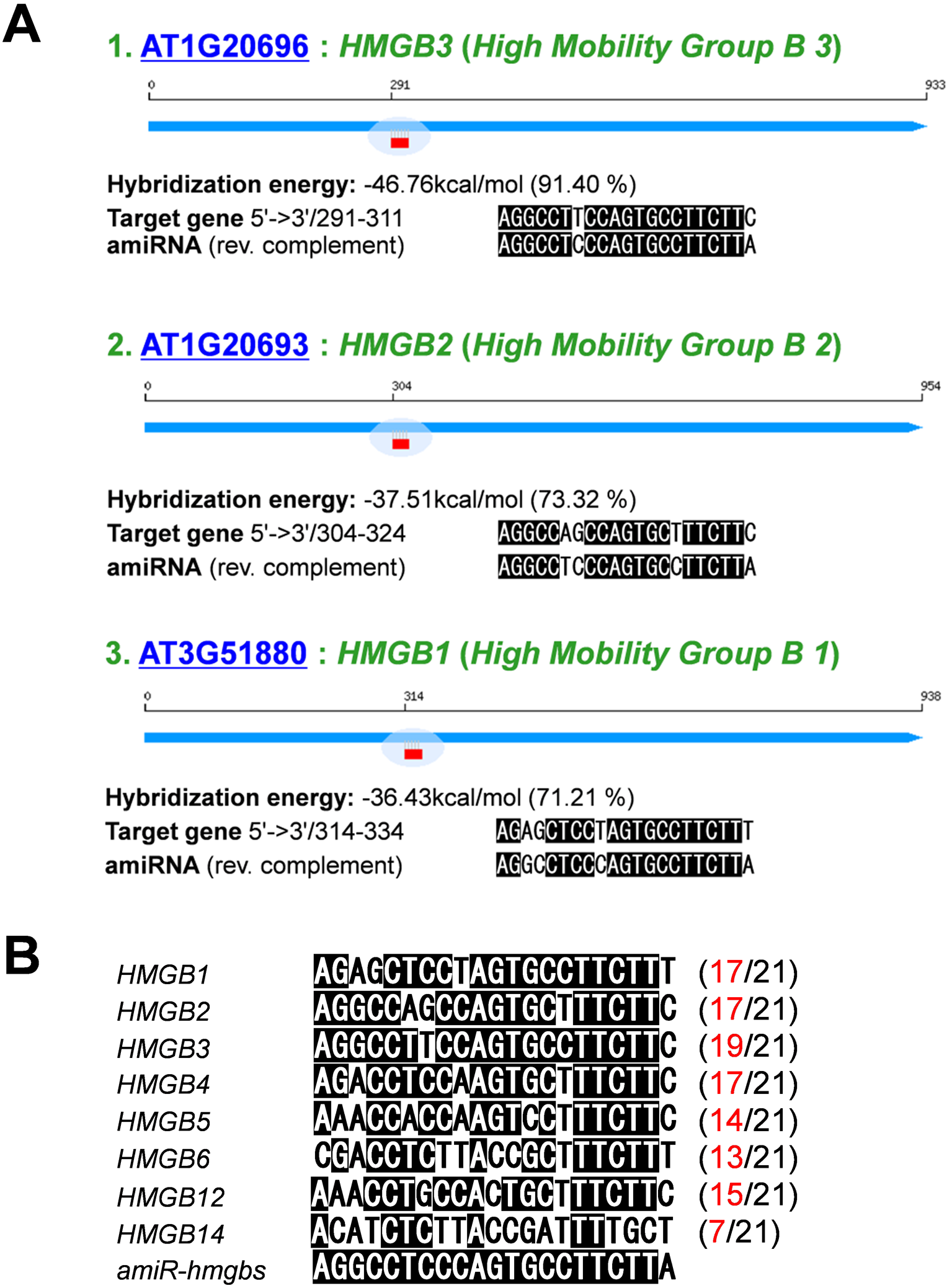

Supplement: S4 Fig — (A) To generate the HMGB3-silencing constructs, an artificial microRNA (amiRNA) was constructed based on a prediction by WMD3-Web MicroRNA designer (http://wmd3.weigelworld.org/) [58,59]. Among the various predicted amiRNAs, an amiR-hmgbs (5’-TAAGAAGGCACTGGGAGGCCT-3’) was selected based on its possible multi-target silencing activity, including HMGB1, HMGB2, and HMGB3. (B) Nucleotide sequence alignment of Arabidopsis HMGBs with amiR-hmgbs. Multiple sequence alignments between Arabidopsis HMGBs and amiR-hmgbs were performed using the Clustal Omega program (Version 1.2.1) [65]. The consensus nucleotide (nt) sequences are presented in white with a black background. The number of identical nucleotides in the sequences of HMGBs and amiR-hmgbs are shown in parentheses in red. HMGB1 (AT3G51880), HMGB2 (AT1G20693), HMGB3 (AT1G20696), HMGB4 (AT2G17560), HMGB5 (AT4G35570), HMGB6 (AT5G23420), HMGB12 (AT5G23405) and HMGB14 (AT2G34450). (TIF) [file ppat.1005518.s004.tif]

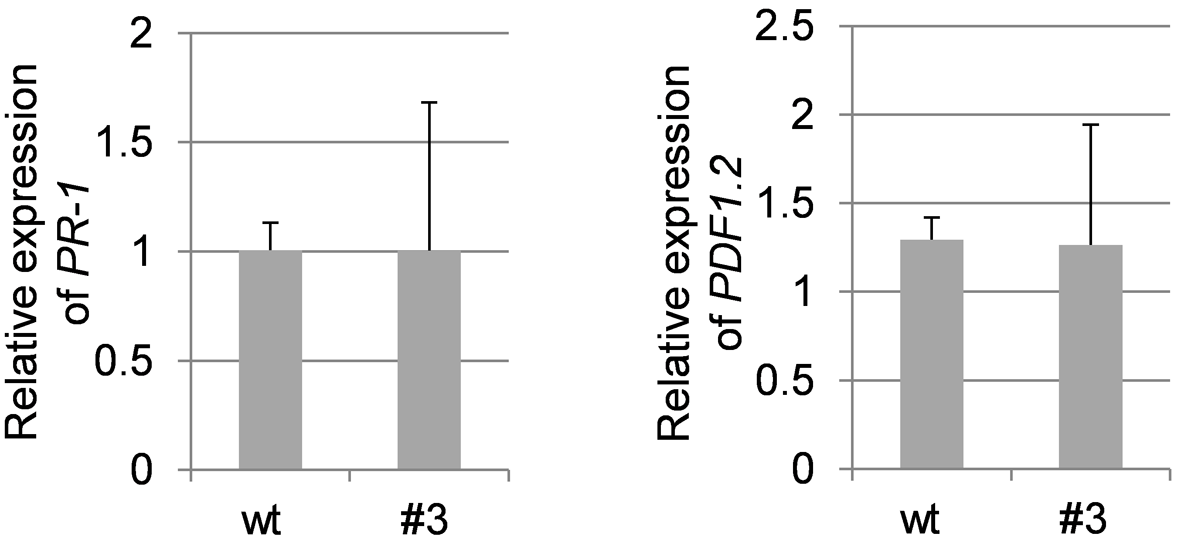

Supplement: S5 Fig — There was no significant difference in the expression of PDF1.2 or PR-1 in untreated wt plants and the amiR-hmgbs transgenic line #3. Expression levels determined by RT-PCR were plotted relative to the expression in untransformed wt leaves, which were set at 1. Data are the mean ± SD (n = 4). (TIF) [file ppat.1005518.s005.tif]

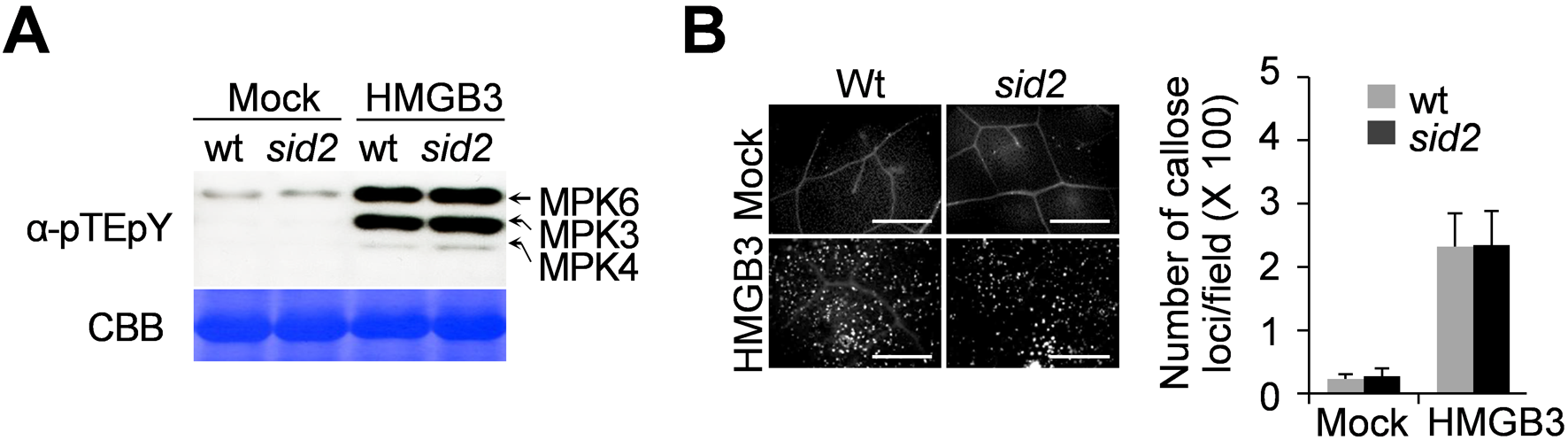

Supplement: S6 Fig — A. MAPK activation in the SA-deficient sid2 mutant Arabidopsis. Leaves were collected 15 min after infiltration with water containing 1 μM HMGB3 for the MAPK activation assay. Activation of MPK3, MPK4, and MPK6 by MAPK kinase-mediated phosphorylation of the TEY sequence was detected with α-pTEpY antibody. Rubisco large subunit protein stained with CBB served as a loading control. B. Callose deposition in Arabidopsis. Leaves were stained with aniline blue 15 h after infiltration with water containing 0.1 μM HMGB3 (= 100 nM) or 2 μg/mL and 20 μg/mL (10X) MBP. Representative pictures are shown in the upper panel. Bars = 100 μm. Data are the mean ± SD (right panel, n = 20). (TIF) [file ppat.1005518.s006.tif]

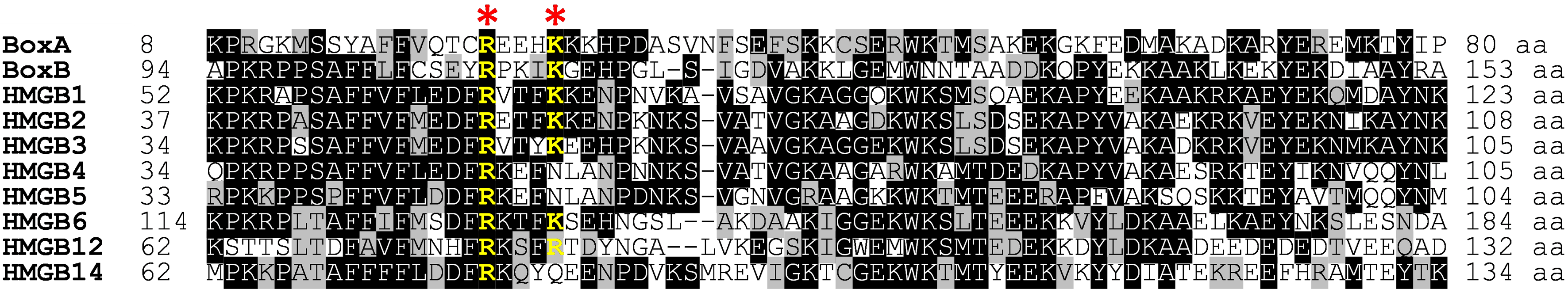

Supplement: S7 Fig — Amino acid sequence alignment of HMG boxes from human and Arabidopsis HMGBs. Human HMGB1 (CAG33144) contains two HMG boxes, designated Box A and Box B [24], whereas the Arabidopsis HMGBs contains only one HMG box: HMGB1 (AT3G51880), HMGB2 (AT1G20693), HMGB3 (AT1G20696), HMGB4 (AT2G17560), HMGB5 (AT4G35570), HMGB6 (AT5G23420), HMGB12 (AT5G23405) and HMGB14 (AT2G34450). Multiple sequence alignments were performed using the Clustal Omega program (Version 1.2.1) [65]. Conserved Arg (R) and Lys (K) residues, which are critical for SA binding, are highlighted in yellow and denoted with yellow asterisks [24]. The consensus and similar amino acid sequences are highlighted by black and grey backgrounds, respectively. Dashes indicate spacing in the amino acid sequences required for proper alignment. (TIF) [file ppat.1005518.s007.tif]

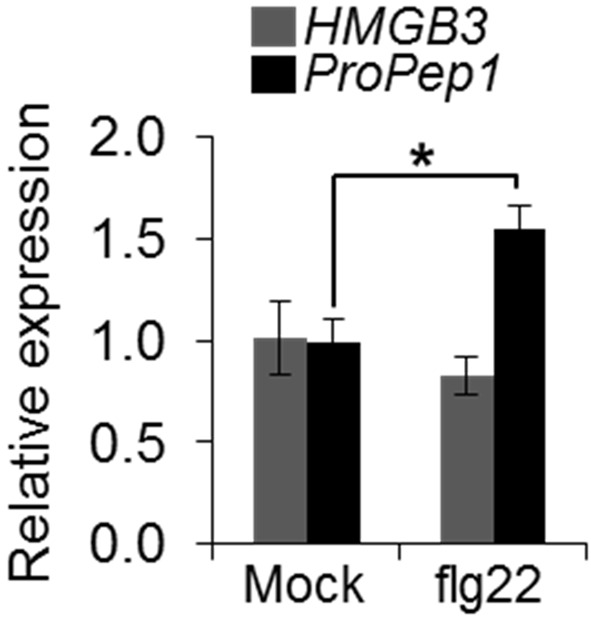

Supplement: S8 Fig — Arabidopsis leaves were infiltrated with water without (Mock) or with 1 μM flg22. Data are the mean ± SD (n = 3). The asterisk indicates significant difference between flg22- and mock-treated leaves (t test, P < 0.05). (TIF) [file ppat.1005518.s008.tif]

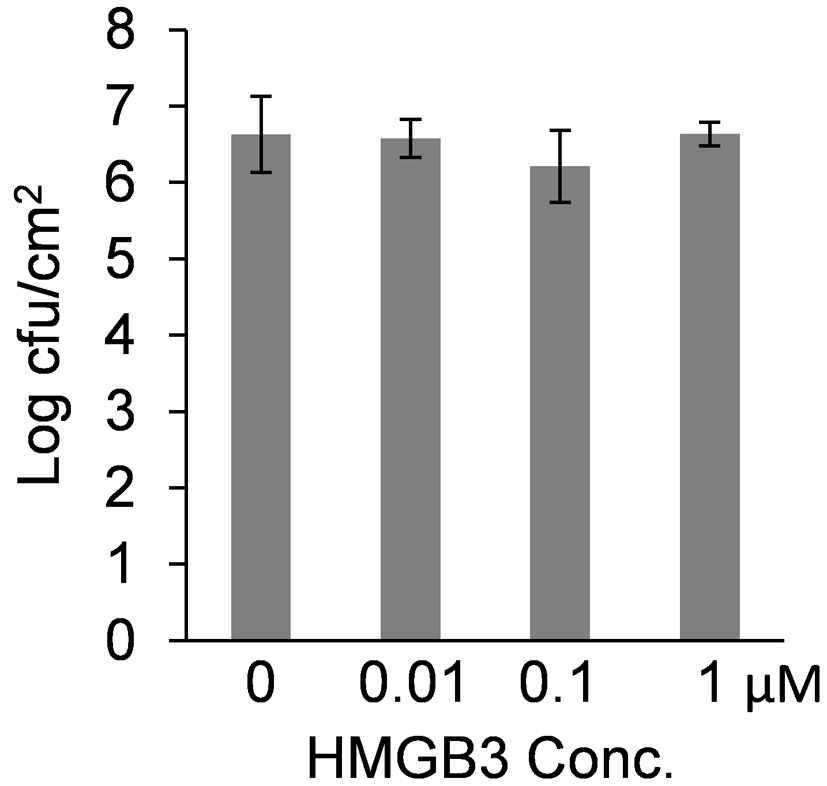

Supplement: S9 Fig — Leaves were infiltrated with the indicated concentrations of HMGB3 one day before P. syringae pv. tomato (Pst) DC3000 inoculation (105 cfu/ml). Bacterial growth was examined 2 days after inoculation. Data are the mean ± SD (n = 5). There were no significant differences between HMGB3- and mock-treated leaves (t test, P < 0.05). (TIF) [file ppat.1005518.s009.tif]
