# Supplementary material for: Activation of Plant Innate Immunity by Extracellular High Mobility Group Box 3 and Its Inhibition by Salicylic Acid
Source: PLoS Pathog. 2016 Mar 23;12(3):e1005518. doi: 10.1371/journal.ppat.1005518 (PMC4805298; doi:10.1371/journal.ppat.1005518)
Supplement: S1 Table — (DOCX) [file ppat.1005518.s010.docx]

| **S1 Table. Oligonucleotides used for cloning in this study** | | |
| --- | --- | --- |
| Transgenic *amiR-hmgbs* cloning | I miR-s | 5’-GATAAGAAGGCACTGGGAGGCCTTCTCTCTTTTGTATTCC-3’ |
|  | II miR-a | 5’-GAAGGCCTCCCAGTGCCTTCTTATCAAAGAGAATCAATGA-3’ |
|  | III miR*s | 5’-GAAGACCTCCCAGTGGCTTCTTTTCACAGGTCGTGATATG-3’ |
|  | IV miR*a | 5’-GAAAAGAAGCCACTGGGAGGTCTTCTACATATATATTCCT-3’ |
|  | A | 5’-CTGCAAGGCGATTAAGTTGGGTAAC-3’ |
|  | B | 5’-GCGGATAACAATTTCACACAGGAAACAG-3’ |
| *HMGB3*-OX cloning | SalI *HMGB3* ORF F | 5’-GTCGAGATGAAAGGAGCTAAATCTAAGGC-3’ |
|  | SacI *HMGB3* ORF R | 5’-GAGCTCCTACCACAACTTCAACTTCTTAATCATCG-3’ |
| Wild-type and R50A  /K54A Mutant *HMGB3* cloning | *HMGB3* ORF F | 5’-ATGAAAGGAGCTAAATCTAAGGCTG-3’ |
|  | *HMGB3* ORF R | 5’-CTACCACAACTTCAACTTCTTAATCATCG-3’ |
|  | *HMGB3* R50A F | 5’-GCTGTGACGTACAAGGAGGAGC-3’ |
|  | *HMGB3* R50A R | 5’-ACGTCACAGCGAAATCTTCCATGAAAACG-3’ |
|  | *HMGB3* K54A (in R50A) F | 5’-GCGGAGGAGCACCCTAAGAACAAG-3’ |
|  | *HMGB3* K54A (in R50A) R | 5’-GCTCCTCCGCGTACGTCACAGCGAAATCTTC-3’ |
