# Supplementary material for: Activation of Plant Innate Immunity by Extracellular High Mobility Group Box 3 and Its Inhibition by Salicylic Acid
Source: PLoS Pathog. 2016 Mar 23;12(3):e1005518. doi: 10.1371/journal.ppat.1005518 (PMC4805298; doi:10.1371/journal.ppat.1005518)
Supplement: S2 Table — (DOCX) [file ppat.1005518.s011.docx]

| **S2 Table. Oligonucleotides used for real-time qPCR in this study** | |
| --- | --- |
| *WRKY33* | 5’-TGTCGTGTGATGCTCTCTCC-3’ |
|  | 5’-GAAACAAATGGTGGGAATGG-3’ |
| *WRKY53* | 5’-ACCGTTGGATTGAACCAGTC-3’ |
|  | 5’-GCGACAAGACACCAGAGTCA-3’ |
| *WRKY70* | 5’-GGAAGAAGACAATCCTCATCGT-3’ |
|  | 5’-CGTTTTCCCATTGACGTAACT-3’ |
| *PDF1.2* | 5’-CGTAACAGATACACTTGTGTGC-3’ |
|  | 5’-CTTATCTTCGCTGATCTTGT-3’ |
| *PR-1* | 5’-GTATGGCTTCTCGTTCACAT-3’ |
|  | 5’-CTAAGAGGCAACTGCAGACT-3’ |
| *PR-2* | 5’-GCTTCCTTCTTCAACCACACAGC-3’ |
|  | 5’-CGTTGATGTACCGGAATCTGAC-3’ |
| *HMGB1* | 5’-GTTGATGACAGAAAGGTGGGAAA-3’ |
|  | 5’-CATTTCTGCCCTCCAGCTTT-3’ |
| *HMGB2* | 5’-AGGAGCTCCAAGCTCTCTGTG-3’ |
|  | 5’-GGACTTCCACTTGTCTCCAGC-3’ |
| *HMGB3* | 5’-GAAACCAGGAGCACAAAGCTC-3’ |
|  | 5’-CTTGCGCTTGTCAGCCTTAG-3’ |
| *HMGB4* | 5’-CGCTACTGTTGGTAAGGCTGC-3’ |
|  | 5’-TCAATCGTCGTCTTCTGCCT-3’ |
| *HMGB5* | 5’-CCAAACGGAAGTTGAATCGAGG-3’ |
|  | 5’-TTCCAGCTGCTCTACCAACATTA-3’ |
| *HMGB6* | 5’-GGAGACACAAGCAGAGGCTA-3’ |
|  | 5’-ACCGATCTTTGCAGCATCCTT-3’ |
| *HMGB12* | 5’-GGAGGCTCTAGTCGGAACTT-3’ |
|  | 5’-CAATCTCGCGCATGGACTTG-3’ |
| *HMGB14* | 5’-TTTTCGCTCGCTGTGAAGAC-3’ |
|  | 5’-ACTTCCACATTTCCCAACCGA-3’ |
| *Tubulin4* | 5’-GTCCAGTGTCTGTGATATTGCACC-3’ |
|  | 5’-TTACGAATCCGAGGGAGCCATTG-3’ |
| *B. cinerea Actin* | 5’-attgctcgtgttgacatggct-3’ |
|  | 5’-tacgatcggagatacctgggt-3’ |
